# SOX1 promotes differentiation of nasopharyngeal carcinoma cells by activating retinoid metabolic pathway

**DOI:** 10.1038/s41419-020-2513-1

**Published:** 2020-05-07

**Authors:** Xin-Xing Lei, Yun Liu, Jin-Xing Wang, Qian Cai, Min Yan, Hui-Ping He, Quentin Liu, Zi-Jie Long, Zhong Guan

**Affiliations:** 1 Sun Yat-sen University Cancer Center, State Key Laboratory of Oncology in South China, 510060 Guangzhou, China; 2 Department of Otorhinolaryngology, Sun Yat-sen Memorial Hospital, Sun Yat-sen University, 510120 Guangzhou, China; 3 Department of Hematology, The Third Affiliated Hospital, Sun Yat-sen University, Institute of Hematology, Sun Yat-sen University, 510630 Guangzhou, China

**Keywords:** Cancer metabolism, Cancer therapy

## Abstract

Undifferentiation is a key feature of nasopharyngeal carcinoma (NPC), which presents as a unique opportunity for intervention by differentiation therapy. In this study, we found that SOX1 inhibited proliferation, promoted differentiation, and induced senescence of NPC cells, which depended on its transcriptional function. RNA-Seq-profiling analysis showed that multiple undifferentiated markers of keratin family, including KRT5, KRT13, and KRT19, were reduced in SOX1 overexpressed NPC cells. Interestingly, gene ontology (GO) analysis revealed genes in SOX1 overexpressed cells were enriched in extracellular functions. The data of LC/MS untargeted metabolomics showed that the content of retinoids in SOX1 overexpressed cells and culture medium was both higher than that in the control group. Subsequently, we screened mRNA level of genes in retinoic acid (RA) signaling or metabolic pathway and found that the expression of UDP-glucuronosyltransferases was significantly decreased. Furtherly, UGT2B7 could rescue the differentiation induced by SOX1 overexpression. Inhibition of UGTs by demethylzeylasteral (T-96) could mimic SOX1 to promote the differentiation of NPC cells. Thus, we described a mechanism by which SOX1 regulated the differentiation of NPC cells by activating retinoid metabolic pathway, providing a potential target for differentiation therapy of NPC.

## Introduction

Nasopharyngeal carcinoma (NPC) is endemic in South-Eastern Asia, and northern Africa. At present, the main treatment approaches for NPC include radiotherapy, chemotherapy, and targeted therapy. Radiation therapy is the first choice for the treatment of NPC. However, it is inevitable that nasopharyngeal mucosa and tissue will be damaged after radiotherapy^1,2^. Thus, more effective and less toxic regimens are needed to offer new opportunities for treating NPC. Recently, remarkable achievements have been made by differentiation therapy in the treatment of some types of tumors^3,4^. 95% of NPC is pathologically undifferentiated squamous cell carcinoma, which make NPC an appropriate model for differentiation therapy.

SOX1 encodes a member of the SOX (SRY-related high mobility group box) family of transcription factors involved in the regulation of embryonic development and determination of the cell fate^5–7^. SOX1 shares similar biochemical properties and functions with SOX2 or other members, but the different affinity to target genes, post-transcriptional modifications and interactions with other co-factors lead to different functions in distinct biological contexts^8–10^. SOX proteins bind sequence-specifically to DNA by a high-mobility group (HMG) domain that allows them to function as transcription factors^8,11^. Gene silencing in cancer is associated with promoter hypermethylation^12^ and SOX1 hypermethylation was detected in primary tumor tissues^13^. SOX1 inhibits cell proliferation, reduces migration and invasion ability, and induces differentiation of tumor cells^14–17^. Recently, we found that SOX1 was associated with decreased expression of undifferentiation markers (KRT13 and KRT19) in NPC cells^16^. However, the mechanism by which SOX1 promotes NPC cell differentiation has not been established.

Retinoic acid (RA), especially all-trans-retinoic acid (ATRA), is the most potent natural metabolite of vitamin A. RA is involved in a variety of biological functions including embryogenesis, cell differentiation, and apoptosis^18,19^. Retinoids induce differentiation and/or apoptosis in tumor cells and show anti-proliferative and anti-oxidant activity, which have great potential as therapeutic or preventive agents. The distribution and level of RA in embryonic tissues are tightly controlled by oxidation through retinol and retinal dehydrogenases and by hydroxylation via specific cytochrome P450 family 26 enzymes (CYP26s)^20,21^.

UDP-glucuronosyltransferases (UGTs) are phase II metabolism isoenzymes that are found to be active in liver, kidneys, epithelial cells of the lower gastrointestinal tract and brain^20,22^. Although the characterizations of UGT gene family members were identified, their roles in clearance and homeostasis of endogenous substrates are still insufficiently understood. Previous study proves that retinoids and their analogs are glucuronidated by UGTs^20,23,24^. Excess retinoids can also inhibit the expression of UGTs, indicating the existence of regulatory loops and complexity^25^.

In the present study, we showed that overexpression of SOX1 promoted NPC cells to differentiate via its transcriptional function. These new data also established a mechanism that retinoids were accumulated by SOX1, contributing to the differentiation of NPC cells, which offered a novel approach for NPC differentiation therapy.

## Materials and methods

### Plasmid constructs

The plasmids encoding human SOX1 (wild type, WT), mutant SOX1 (SOX1^mut_2^), V5-tagged SOX1, ΔHMG SOX1, and Δ246-391 SOX1 were generated by PCR amplification and subcloned into the pLVX-TRE3G expression vector; UGT1A6 and UGT2B7 were subcloned into pLVX expression vector; promoters of UGT1A6 and UGT2B7 were subcloned into pGL3-basic vector using the ClonExpress II One Step Cloning Kit or ClonExpress MultiS One Step Cloning Kit (Vazyme) according to the manufacturer’s instructions. The new plasmid was named pLVX-TRE-(X), where X stood for SOX1 (wild type, WT), mutant SOX1 (SOX1^mut_2^), V5-tagged SOX1, ΔHMG SOX1, or Δ246-391 SOX1. The primers used for gene cloning and plasmid construction are listed in Supplementary Table S1.

### Lentiviral production, infections, and cell line generation

Lentivirus was produced by transient transfection by Lipofectamine 2000 (Invitrogen) in human embryonic kidney (HEK) 293T cells using with a second-generation lentiviral vector system. All virus-containing medium was mixed with 8 μg/mL polybrene (Sigma-Aldrich). Virus produced from pLVX-Tet3G plasmid was used to infect wild type HONE1 or CNE2 cells to construct HONE1-Tet-On or CNE2-Tet-On stable cell lines. Cells were selected in 1 mg/mL G418 for at least 2 weeks. Subsequently, HONE1-Tet-On and CNE2-Tet-On cell lines were infected by virus produced from pLVX-TRE-(X) plasmids and selected by 2 μg/mL puromycin for 6 days. HONE1^TRE-SOX1^ and CNE2^TRE-SOX1^ cells infected by virus produced from pLVX-UGT1A6 or pLVX-UGT2B7 plasmids were selected by 12 μg/mL blasticdin for 7 days.

### Cell culture and doxycycline induction

The HONE1 and CNE2 cell lines were obtained from Dr. Chao-Nan Qian (Sun Yat-sen University, Guangzhou, China). Wild type cell lines and their lentiviral-infected stable cell lines were all maintained in RPMI 1640 (Invitrogen) supplemented with 10% fetal bovine serum (FBS, Hyclone). The cells were incubated at 37 °C in a humidified chamber containing 5% CO_2_. HONE1-Tet-On-(X) and CNE2-Tet-On-(X) cell lines were treated with 2 μg/mL doxycycline (Medchem Express) to induce overexpression of SOX1 or its mutant/truncated form.

### Cell viability assay

Cells were seeded onto 96-well plates at an initial density of 2 × 10^3^ cells/well. At specified time points, 10 μL of CCK-8 solution was added to each well of the plate. Then the plate was incubated for another 2 h. Cell viability was determined by scanning with a microplate reader at 450 nm.

### Immunofluorescence staining

Cells were fixed in 4% paraformaldehyde at room temperature for 10 min and permeabilized in 0.5% Triton X-100 in PBS for 10 min. Slides were incubated with the primary antibody (1:200 dilution) overnight. Immune complexes were stained with the secondary antibody conjugated to Alexa-488 or Alexa-546 (Invitrogen, 1:200 dilution) at room temperature for 1 h. Nuclei were stained with DAPI (Sigma-Aldrich) and viewed with an Olympus IX71 microscope. The following antibodies were used as primary antibodies: E-cadherin and V5-tag (Cell Signaling Technology 3195 and 13202, respectively), Ki-67 (Proteintech 27309-1-AP), and SOX1 (GeneTex EPR4766).

### Colony formation assay

Approximately 500 cells were seeded into six-well plates in triplicate and incubated for 8 days. Colonies were stained with crystal violet and viewed.

### Senescence-associated β-galactosidase (SA-β-gal) staining

Cultured cells were washed in PBS and SA-β-gal activity was detected using senescence β-galactosidase staining kit (Beyotime) according to the manufacturer’s direction.

### Cell lysis and western blot analysis

Cells were lysed on ice in RIPA buffer. Protein concentration was determined by using the Bradford dye method. Equal amounts of cell extracts were subjected to electrophoresis in 10% gradient SDS–PAGE gels and then transferred to 0.45 µm PVDF membranes (Millipore) for antibody blotting. The following antibodies were used: V5-tag, p21 Waf1/Cip1, Rb, Phospho-Rb (Ser780), Phospho-mTOR (Ser2448), Phospho-p70 S6 Kinase (Thr389), Phospho-p70 S6 Kinase (Ser371), and Phospho-4E-BP1 (Thr37/46) (Cell Signaling Technology 13202, 2947, 9309, 9307, 2971, 9205, 9208 and 2855, respectively), SOX1 (GeneTex EPR4766), KRT19 (Abcam EP1580Y), KRT13, mTOR, p70 S6 Kinase and 4E-BP1, (Epitomics 2713-1, 1612-1, 1494-1, and 1557-1, respectively), KRT5, CDK4, CDK6, c-Myc, PPARγ, and β-actin (Proteintech 66727-1-Ig, 11026-1-AP, 14052-1-AP, 10828-1-AP, 16643-1-AP, and 60008-1-Ig, respectively), UGT1A6 and UGT2B7 (Signalway Antibody 43176 and C32390, respectively). Horseradish peroxidase-conjugated goat anti-mouse or goat anti-rabbit IgG (Pierce) was used as a secondary antibody. Proteins were visualized with Immobilon Western Chemilum HRP Substrate (Millipore).

### RNA-Seq

RNA-Seq data generation and normalization were performed on an Illumina HiSeq™ PE150 system by the Novogene Bioinformatics Technology Co., Ltd. (Beijing, China).

### Quantitative real-time PCR (RT-PCR)

Total RNA was extracted by using HiPure Total RNA Kits (Magen), which was used to generate cDNA by using One-Step RT-PCR SuperMix (TransScript). Quantitative RT-PCR was performed using ChamQ SYBR qPCR Master Mix (Vazyme) as recommended by the manufacturer. The primers used were listed in Supplementary Tables S2–4. ACTB was used as the internal control.

### Gene set enrichment analysis (GSEA)

GSEA was performed using hallmark gene sets from the Molecular Signatures Database (MSigDB). RNA-Seq expression data sets were loaded into GSEA 3.0 software. 10,000 permutations were done and finally a list of gene set ranks with information of normalized enrichment scores (NES), *P*-value and FDR-*q*-value was obtained. The top significant gene sets were viewed and sorted.

### Conditional medium (CM) preparation

For collecting CM of NPC cells, HONE1-Tet-On SOX1 cells were cultured in RPMI 1640 supplemented with 10% FBS. At a 50% confluence, medium was discarded and cells were incubated with fresh RPMI 1640 (10% FBS) with or without 2 μg/mL doxycycline for 48 h. The CM was then collected, filtered using a 0.22 μm filter, and stored at −80 °C until use.

### Metabolic profiling by LC–MS

LC–MS analysis was performed on an ACQUITY UHPLC system (Waters Corporation) coupled with an AB SCIEX Triple TOF 5600 System (AB SCIEX) equipped with an electrospray ionization source, at both positive and negative ion modes. Each group contains five replicative samples. The stability of LC–MS analytical systems was evaluated using the pooled QC samples at regular intervals (every five samples). The LC–MS data were exported as .wiff files and converted to .mzXML files using msconvert software (ProteoWizard tool). The files were then uploaded on XCMS database (https://xcmsonline.scripps.edu) for further analysis according to XCMS instruction.

### Cell cycle, apoptosis, and dual-luciferase reporter assay

Cells were collected and fixed in 70% ethanol at −20 °C overnight and then washed and resuspended in FxCycle™ PI/RNase Staining Solution (Invitrogen). After incubating in the dark for 30 min at room temperature, cell cycles were analyzed by flow cytometry.

Cells were collected and resuspended in the binding buffer and then Annexin-V-FITC and PI (Invitrogen) were added to the cells according to the protocol. The cells were then incubated for 15 min in the dark and subjected to flow cytometry for apoptosis analysis.

Luciferase activity in cell lysates was determined by the dual-luciferase reporter assay system (Promega). Firefly luciferase data for each sample were normalized against Renilla luciferase activity.

### Statistical analysis

Each experiment was performed in triplicate and repeated at least three times. Unless otherwise indicated, data were presented as mean ± SD of three independent experiments. Statistics were calculated by Prism Graphpad software (version 7.0). Differences among variables were assessed by two-tailed Student’s *t*-tests and one-way or two-way ANOVA with multiple comparisons tests. A *P*-value < 0.05 was considered statistically significant (*P < 0.05, **P < 0.01, ***P < 0.001, ****P < 0.0001).

## Results

### Overexpression of SOX1 promotes the differentiation of NPC cells

**Fig. 1:**
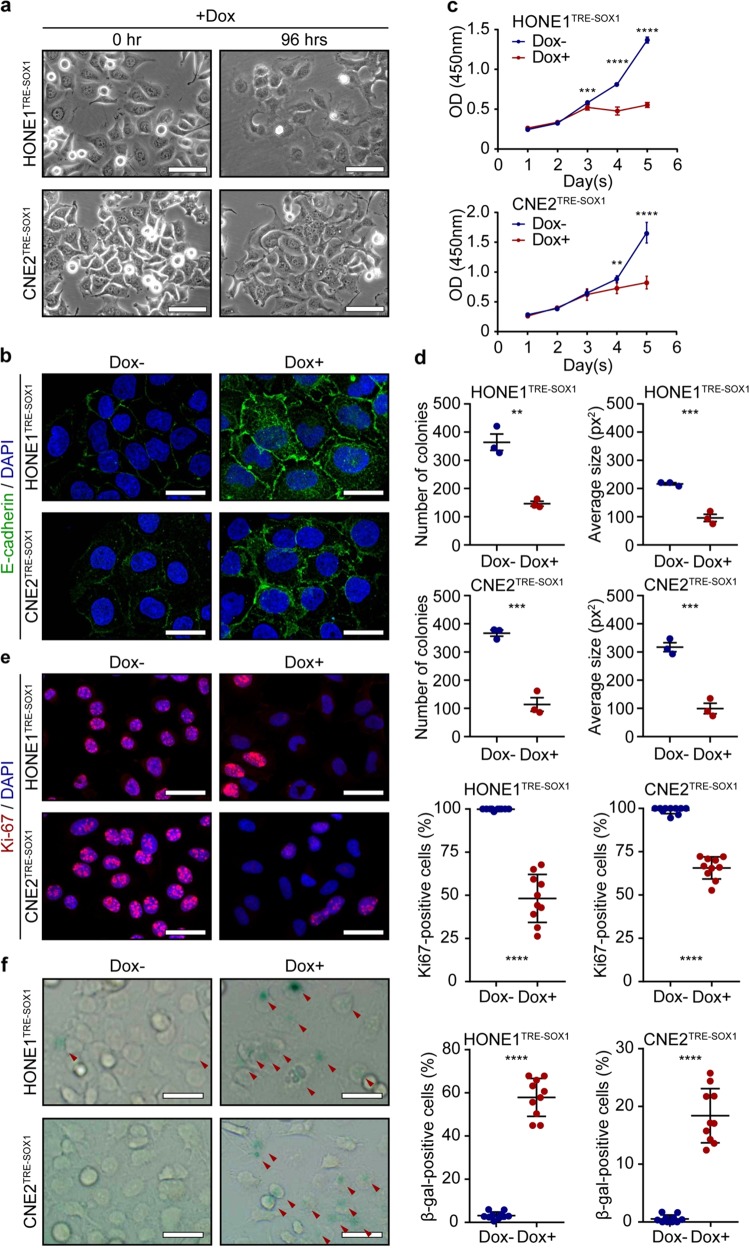
SOX1 overexpression promotes differentiation of NPC cells. **a** Morphology of HONE1^TRE-SOX1^ and CNE2^TRE-SOX1^ cells with doxycycline treatment for 72 h. Scale bar = 50 µm. **b** Confocal immunofluorescence for E-cadherin (green) and DAPI (blue) in HONE1^TRE-SOX1^ and CNE2^TRE-SOX1^ cells with or without doxycycline treatment for 96 h. Scale bar = 30 μm. **c** Cell viability of HONE1^TRE-SOX1^ and CNE2^TRE-SOX1^ cells with (red) or without (blue) doxycycline treatment by CCK-8 assay. All data represent the mean ± SD (*n* = 4, **P < 0.01, ***P < 0.001, ****P < 0.0001). **d** Colony formation assay of HONE1^TRE-SOX1^ and CNE2^TRE-SOX1^ cells with or without doxycycline treatment for 8 days. Dot plots show number and average size of colonies calculated by imageJ software. All data represent the mean ± SD (*n* = 3, **P < 0.01, ***P < 0.001). **e** Confocal immunofluorescence (left panel) for Ki-67 (red) and DAPI (blue) in HONE1^TRE-SOX1^ and CNE2^TRE-SOX1^ cells with or without doxycycline treatment. Scale bar = 50 μm. Dot plots (right panel) show quantification of the frequency of Ki-67-positive cells in each vision. All data represent the mean ± SD (*n* = 10, ****P < 0.0001). **f** SA-β gal staining (left panel) of HONE1^TRE-SOX1^ and CNE2^TRE-SOX1^ cells with or without doxycycline treatment. Red arrows represent SA-β gal-positive cells. Scale bar = 50 μm. Dot plots (right panel) show quantification of the frequency of SA-β gal-positive cells in each vision. All data represent the mean ± SD (*n* = 10, ****P < 0.0001).

To determine whether SOX1 was required for the development of differentiated NPC status, stable clones overexpressing SOX1 were selected from two poorly differentiated NPC cell lines (HONE1 and CNE2) using the Tet-ON system. Expression of SOX1 was induced by doxycycline compared to the cells without doxycycline. Induction of SOX1 resulted in morphologic changes histologically characterized as slender and fusiform phenotype in HONE1 and CNE2 cells (Fig. 1a). E-cadherin was up-regulated in HONE1 and CNE2 cells with SOX1 overexpression (Fig. 1b). In addition, overexpression of SOX1 attenuated cell proliferation and colony formation (Fig. 1c–e, Supplementary Fig. 1). Besides, the proportion of senescence-associated β-gal-positive cells was increased following SOX1 overexpression (Fig. 1f). The results indicated that SOX1 promoted the differentiation of NPC cells.

### SOX1 promoting NPC cell differentiation depends on its transcriptional function

**Fig. 2:**
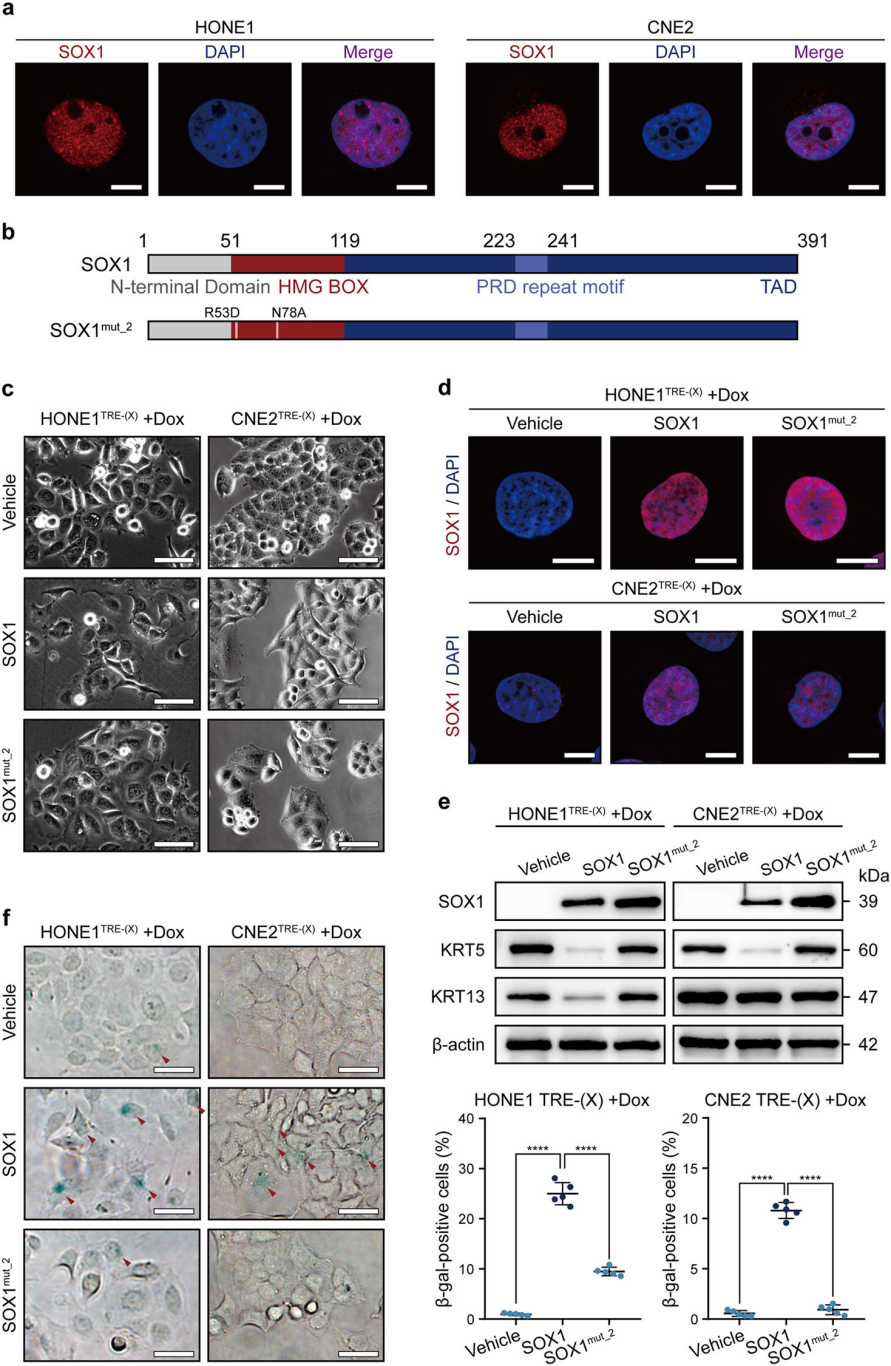
HMG box is indispensable for SOX1 to promote NPC cell differentiation. **a** Confocal immunofluorescence for SOX1 (red) and DAPI (blue) in HONE1 and CNE2 cells. Fluorescence images are respectively merged to display location of SOX1 (red). Scale bar = 4 µm. **b** Schematics of full length and mutated SOX1 proteins used in this study. **c** Morphology of HONE^TRE-(X)^ and CNE2^TRE-(X)^ cells (X stand for vehicle, wild type SOX1 or mutant SOX1) under doxycycline treatment for 3 days. Scale bar = 50 µm. **d** Confocal immunofluorescence for SOX1 (red) and DAPI (blue) in HONE^TRE-(X)^ and CNE2^TRE-(X)^ cells under doxycycline treatment for 3 days. Scale bar = 4 μm. **e** Western blot analysis of SOX1, KRT5, KRT13, and β-actin expression in HONE^TRE-(X)^ and CNE2^TRE-(X)^ SOX1 cells under doxycycline treatment for 3 days. β-actin was used as a control. **f** SA-β gal staining (left panel) of HONE^TRE-(X)^ and CNE2^TRE-(X)^ cells under doxycycline treatment for 7 days. Red arrows represent SA-β gal-positive cells. Scale bar = 50 μm. Dot plots (right panel) show quantification of the frequency of SA-β gal-positive cells in each vision. All data represent the mean ± SD (*n* = 5, ****P < 0.0001).

The mechanism underlying the role of SOX1 during the cellular differentiation process of NPC remained largely elusive. Previous study showed that SOX family members regulated developmental events not only by acting as direct transcriptional regulators, but also by forming protein–protein interactions and acting as either coactivators or co-suppressors^8^. To reveal the underlying mechanism, we firstly detected SOX1 expression in HONE1 and CNE2 cells and found that SOX1 was localized in the nucleus (Fig. 2a). Then, two truncated SOX1 expression vectors were also made, namely ΔHMG SOX1 (SOX1 without the N-terminus) and Δ246-391 SOX1 (SOX1 without the C-terminus), together with a full-length SOX1 expression vector (V5-tagged SOX1) (Supplementary Fig. 2a). All of the vectors were transferred to HONE1 and CNE2, respectively. The cellular morphology and related differentiation protein makers of two truncated SOX1 expressed NPC cells were similar to the vehicle group (empty vector) (Supplementary Fig. 2b–d). Moreover, two truncated SOX1 expression did not induce cell senescence (Supplementary Fig. 2e). These data showed that loss of the HMG box and the C-terminus (transcription activation domain) of SOX1 significantly reduced the ability of SOX1 to promote NPC cell differentiation. HMG box is essential for binding with DNA. The homology of HMG box of SOX1 was highly similar with other SOX subfamily members, especially SOX2 (Supplementary Fig. 3). Thus, we mutated two amino acids (Arg53 to Asp and Asn78 to Ala) in the DNA-binding sites of SOX1 according to SOX2 crystal structure (Supplementary Fig. 4, Fig. 2b). The expression of mutant SOX1 was still localized in the nucleus as detected by immunofluorescence staining (Fig. 2d). The cellular morphology and related differentiation protein makers of the mutant SOX1 expressed NPC cells were similar to the vehicle group (empty vector) (Fig. 2c, e). Moreover, mutant SOX1 expression did not induce cell senescence (Fig. 2f). The data showed that mutant of the HMG box in SOX1 significantly impaired the ability of SOX1 to promote NPC cell differentiation. Taken together, these findings indicated that SOX1 induced NPC cell differentiation attributing to its transcriptional function.

### SOX1 induces a global alteration of keratins

**Fig. 3:**
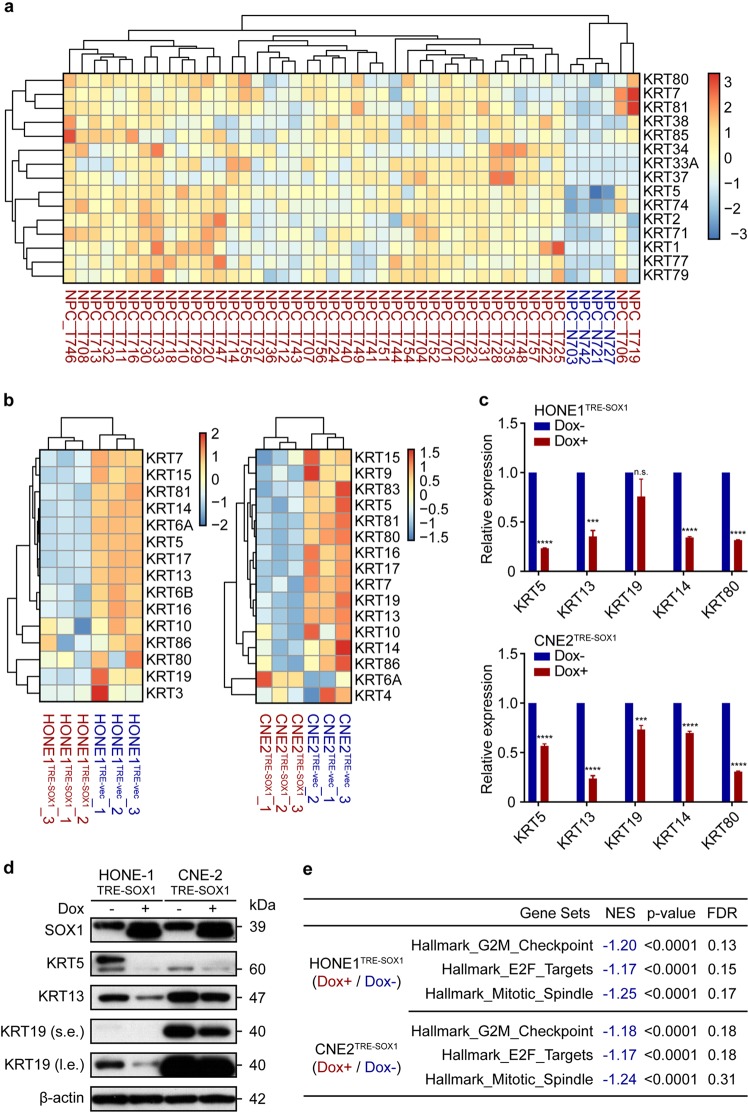
Screening of NPC cells undifferentiated markers in keratin gene family. **a** RNA-Seq analysis displays heat map of keratins gene family expressed in 42 Chinese NPC patients and 4 non-NPC tissues from GEO database (GSE68799). mRNA intensities were rlog transformed and are displayed as colors ranging from red to blue. Both rows and columns are clustered using correlation distance and average linkage. **b** RNA-Seq analysis shows heat map of keratin gene family expressed in HONE1^TRE-SOX1^ (left) and CNE2^TRE-SOX1^ (right) cells under doxycycline treatment for 4 days. mRNA intensities were rlog transformed and are displayed as colors ranging from red to blue. Both rows and columns are clustered using correlation distance and average linkage. **c** RT-PCR analysis of keratin genes in HONE1^TRE-SOX1^ (upper) and CNE2^TRE-SOX1^ (lower) cells with (red) or without (blue) doxycycline treatment for 48 h. Data are normalized by the amount of ACTB mRNA and represent mean ± s.e.m. (*n* = 3, n.s.: *P* > 0.05, ***P < 0.001, ****P < 0.0001). **d** Western blot analysis of keratin proteins, SOX1, and β-actin in HONE1^TRE-SOX1^ and CNE2^TRE-SOX1^ cells with or without doxycycline treatment for 96 h. β-actin was used as a loading control. s.e.: short exposure, l.e.: long exposure. **e** GSEA of SOX1 (Dox+) vs. control (Dox−) in HONE1^TRE-SOX1^ and CNE2^TRE-SOX1^ cells using ‘Hallmark G2M Checkpoint’, ‘Hallmark E2F Targets’, and ‘Hallmark mitotic spindle’ gene sets. NES normalized enrichment score, FDR false discovery rate.

Although it has been reported that keratins were used as indicators of differentiation, there was no systematic studies on how the family of keratins altered between differentiated and undifferentiated NPC. Here, we analyzed RNA-Seq data of 42 cases of carcinoma tissues as well as 4 cases of normal tissues from GEO database. A global high expression of keratins in NPC tissues was found (Fig. 3a). In order to understand whether the expression of SOX1 in NPC cells could influence the expression of keratins, we treated HONE1^TRE-SOX1^ and CNE2^TRE-SOX1^ with or without doxycycline for 4 days, and then RNA-Seq was performed. It showed that the induction of SOX1 greatly reduced the expression of keratins (Fig. 3b). Moreover, RT-PCR and western blot assay were performed to verify the expression patterns of keratins at mRNA and protein level (Fig. 3c, d). The results showed SOX1 decreased the expression of keratins, including KRT5, KRT13, and KRT19, which were basically consistent with the results of RNA-Seq data. Thus, these data suggested that low expression of SOX1 was associated with high expression of keratins in NPC. GSEA displayed that genes were enriched in regulation of G2/M checkpoint, E2F targets and mitotic spindle after SOX1 overexpression (Fig. 3e). Moreover, as cell differentiation usually require G0/G1 arrest, and E2F targets were enriched after SOX1 overexpression, cell cycle distribution was then detected. Results showed that SOX1 induced cell cycle G0/G1 phase arrest (Supplementary Fig. 5a, b), but did not promote obvious apoptosis (Supplementary Fig. 6). GSEA analysis revealed that SOX1 downregulated Myc target genes and inhibited mTOR1 pathway. SOX1 could significantly decrease the expression of c-Myc and mTOR1 downstream proteins in NPC cells (Supplementary Fig. 5c–f).

### SOX1 promotes differentiation of NPC cells by activating retinoid pathway

**Fig. 4:**
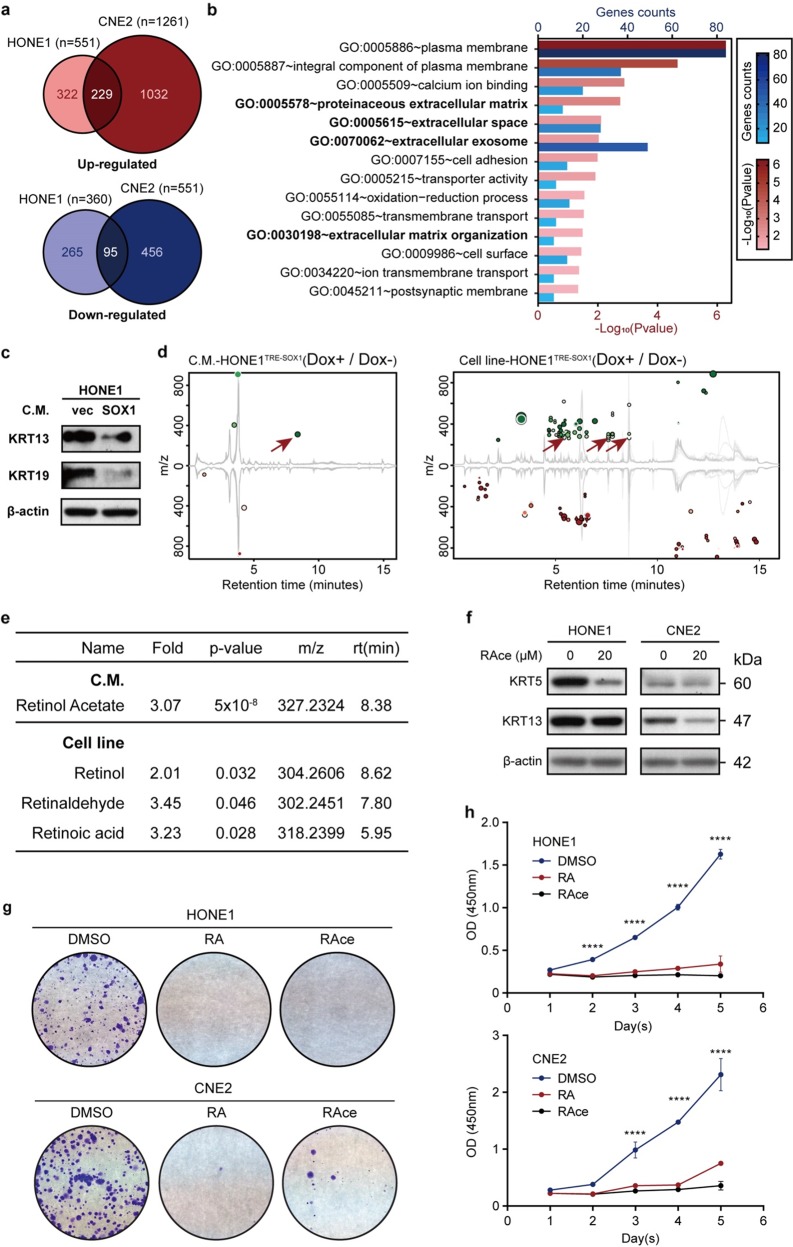
Untargeted LC/MS-based metabolomics reveals enrichment of retinoid pathway in SOX1 overexpressed cells. **a** Significantly up-regulated (upper) or down-regulated (lower) mRNAs (|fold-change | ≥2 and *P* < 0.05) in HONE1^TRE-SOX1^/CNE2^TRE-SOX1^ (Dox+) compared to corresponding control groups (Dox−) were shown by venn diagrams. **b** GO analysis of the differently expressed genes. Blue column represents the total number of genes annotated with each GO term, while red is −log_10_ of *P* value. **c** Western blot analysis of keratin proteins and β-actin of wild type HONE1 cultured with conditional-media (CM) of HONE1^TRE-SOX1^ cell with (SOX1) or without (vec) doxycycline treatment for 48 h. β-actin was used as a loading control. **d** Differential feature plot for CM and cells of HONE1^TRE-SOX1^ with or without doxycycline treatment by LC–MS untargeted metabolomics. Only features that are dysregulated (*P*-value ≤ 0.05, fold change ≥ 1.5) are displayed. Upregulated features are shown in green, while downregulated features in red. The size of each bubble corresponds to the log fold change of that feature. The shade of the bubbles corresponds to the magnitude of the *P*-value (the darker the color, the smaller the *P*-value). Red arrows represent metabolites in retinoid pathway. **e** Summary of fold change, *P*-value, mass-to-charge ratio (*m*/*z*), and retention time (rt) of metabolites in retinoid pathway screened in **d**. **f** Western blot analysis of KRT5, KRT13, and β-actin of wild type HONE1 and CNE2 cells with or without RAce treatment for 72 h. β-actin was used as a loading control. **g** Colony formation assay of wild type HONE1 and CNE2 cells with vehicle, RA (10 μM), or RAce (10 μM) treatment for 8 days. **h** Cell viability of wild type HONE1 and CNE2 cells with (red) or without (blue) doxycycline treatment by CCK-8 assay. All data represent the mean ± SD (*n* = 4, ****P < 0.0001).

RNA-Seq displayed significantly 229 up-regulated genes and 95 down-regulated genes in HONE1^TRE-SOX1^ and CNE2^TRE-SOX1^ (Dox+) compared to corresponding control groups (Dox−) (Fig. 4a). Gene ontology (GO) analysis of differentiated cells showed that genes were enriched in extracellular activities, suggesting that the extracellular components of differentiated cells might be distinct from undifferentiated cells (Fig. 4b). To verify our hypothesis, we treated HONE1 cells with CM obtained from HONE1^TRE-SOX1^ cell culture medium (Dox+). Surprisingly, CM-induced alteration of KRT13 and KRT19 (Fig. 4c). Then LC–MS untargeted metabolomics was used to analyze the composition of the CM and cells of HONE1^TRE-SOX1^ with or without doxycycline treatment. The results showed that the content of retinoids increased in both CM and cells with doxycycline treatment (Fig. 4d, e). To determine whether cell differentiation was due to elevated retinoids level, NPC cells were treated with retinol acetate (RAce) and subjected to detection of protein levels. The expression of KRT5 and KRT13 was decreased significantly, which demonstrated that retinoid metabolic pathway was involved in NPC cellular differentiation (Fig. 4f). Moreover, RA/RAce remarkably inhibited colony formation and cell viability of NPC cells (Fig. 4g, h). These data suggested that SOX1 promoted differentiation of NPC cells by activating retinoid pathway.

### UGT2B7 disrupts SOX1 to promote differentiation of NPC cells

**Fig. 5:**
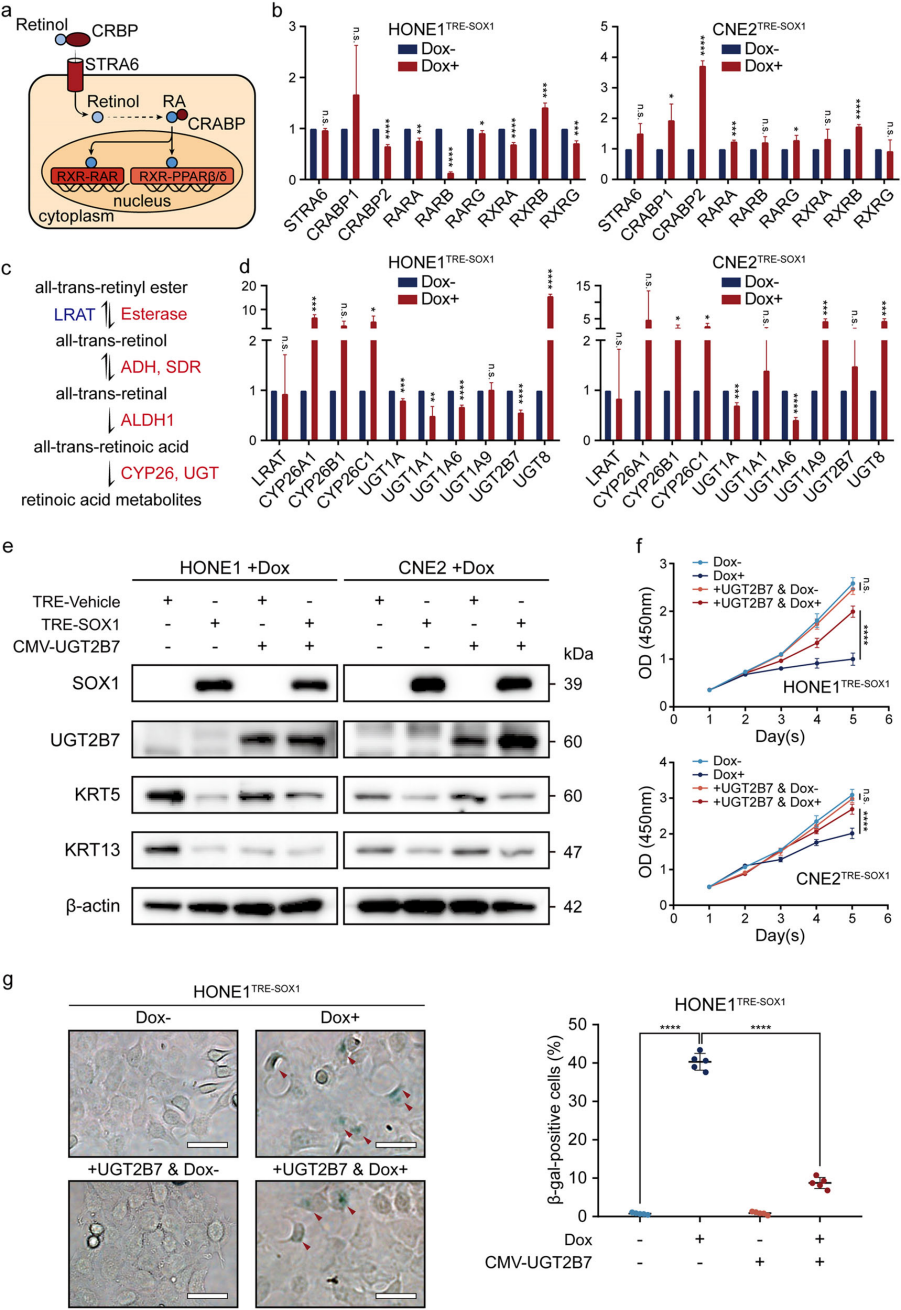
SOX1 deregulates UGTs expression to activate retinoid pathway in NPC cells. **a** A brief overview of retinoic acid signaling pathway. Retinol transports to cells in a complex with CRBP through vitamin A receptor STRA6. In cytoplasm, retinol is oxidized and converted to RA. RA can complex with CRABP1/2 and transports to the nucleus. Following, RA forms a complex with RXR–RAR or RXR–PPARβ/δ heterodimers and binds to DNA of retinoic acid response element (RARE) or PPAR response element (PPRE), thereby activating transcription of target genes. **b** RT-PCR analysis of STAR6, CRABP1, CRABP2, RARA, RARB, RARG, RXRA, RXRB, and RXRG genes in HONE1^TRE-SOX1^ (left) and CNE2^TRE-SOX1^ (right) cells with (red) or without (blue) doxycycline treatment for 48 h. Data are normalized by the amount of ACTB mRNA, and compared to the corresponding value for cells without doxycycline treatment. Data represent mean ± s.e.m. (*n* = 3, n.s.: *P* > 0.05, *P < 0.05, **P < 0.01, ***P < 0.001, ****P < 0.0001). **c** A brief overview of all-trans-retinoic acid metabolic pathway. In cells, all-trans-retinol can be converted to all-trans-retinal by alcohol dehydrogenase (ADH) or short-chain dehydrogenase/reductase (SDR), or to all-trans-retinyl esters by lecithin retinol acyltransferase (LRAT). All-trans-retinal can be further converted to all-trans-retinoic acid by aldehyde dehydrogenase (ALDH). Finally, all-trans-retinoic acid is metabolized to inactive retinoids by CYP26s or UGTs. **d** RT-PCR analysis of LRAT, CYP26A1, CYP26B1, CYP26C1, UGT1A (total), UGT1A1, UGT1A6, UGT1A9, UGT2B7, and UGT8 genes in HONE1^TRE-SOX1^ (left) and CNE2^TRE-SOX1^ (right) cells with (red) or without (blue) doxycycline treatment for 48 h. Data are normalized by the amount of ACTB mRNA and represent mean ± s.e.m. (*n* = 3, n.s.: *P* > 0.05, *P < 0.05, **P < 0.01, ***P < 0.001, ****P < 0.0001). **e** Western blot analysis of SOX1, UGT2B7, KRT5, KRT13, and β-actin expression in HONE1^TRE-SOX1^, CNE2^TRE-SOX1^ as well as UGT2B7 overexpressed HONE1^TRE-SOX1^ and CNE2^TRE-SOX1^ cells under doxycycline treatment for 4 days. β-actin was used as a control. **f** Cell viability of HONE1^TRE-SOX1^, CNE2^TRE-SOX1^ as well as UGT2B7 overexpressed HONE1^TRE-SOX1^ and CNE2^TRE-SOX1^ cells with or without doxycycline treatment by CCK-8 assay. All data represent the mean ± SD (*n* = 4, ****P < 0.0001). **g** SA-β gal staining (left panel) of HONE1^TRE-SOX1^ and UGT2B7 overexpressed HONE1^TRE-SOX1^ cells under doxycycline treatment for 7 days. Red arrows represent SA-β gal-positive cells. Scale bar = 50 μm. Dot plots (right panel) show quantification of the frequency of SA-β gal-positive cells in each vision. All data represent the mean ± SD (*n* = 5, n.s.: *P* > 0.05, ****P < 0.0001).

Our data showed that the content of retinoids was increased in differentiated NPC cells due to overexpressed SOX1. Retinoids signaling and metabolism diagrams were drawn to represent how retinol transports to cells and converts to RA (Fig. 5a, c). The content of RA in cells is tightly controlled by numerous enzymes involved in retinoid metabolism. Thus, the mechanism of SOX1 increasing RA accumulation in NPC cells was investigated. RT-PCR was performed to detect the expression of RA signaling pathway-related enzymes or receptors: the RA-inducible gene stimulated by retinoic acid 6 (STRA6), cellular retinoic acid-binding protein 1 (CRABP1), cellular retinoic acid-binding protein 2 (CRABP2), RARs (RARA, RARB, and RARG) and RXRs (RXRA, RXRB, and RXRG). Moreover, lecithin retinol acyltransferase (LRAT), cytochrome P450 family 26 subfamily (CYP26A1, CYP26B1, and CYP26C1), and UDP glucuronosyltransferase family (UGT1A (total), UGT1A1, UGT1A6, UGT1A9, UGT2B7, and UGT8) genes were also detected (Fig. 5b, d). The data showed that SOX1 suppressed several UGT genes expression, including UGT1A6 and UGT2B7 (Fig. 5d). Then dual-luciferase reporter assay revealed that SOX1 did not affect UGT1A6 or UGT2B7 promoters’ transcriptional activity (Supplementary Fig. 7). We continued to overexpress UGT1A6 or UGT2B7 in SOX1 ectopic expressed cells, and found that UGT2B7, but not UGT1A6, could partially rescue the ability of SOX1 to induce NPC cell differentiation (Fig. 5e–g, Supplementary Fig. 8). These data indicated that UGT2B7 could be the target of SOX1. However, RA metabolic network regulated by SOX1 was coordinately balanced by multiple factors, but not only UGT2B7.

**Fig. 6:**
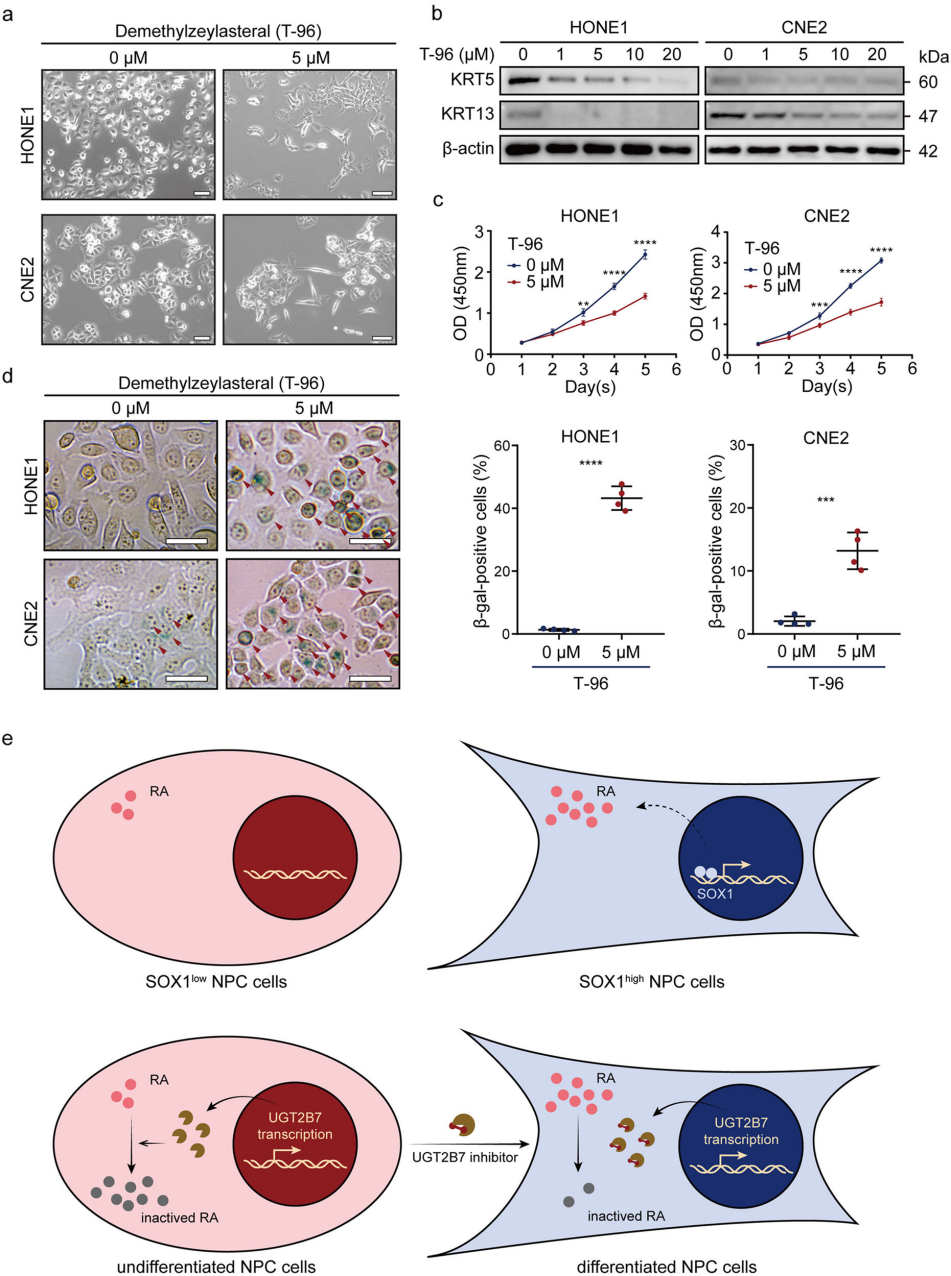
Targeting UGTs promotes differentiation of NPC cells. **a** Morphology of wild type HONE1 and CNE2 cells with or without T-96 treatment for 72 h. Scale bar = 50 µm. **b** Western blot analysis of KRT5, KRT13, and β-actin of wild type HONE1 and CNE2 cells with 0, 1, 5, 10, 20 μM T-96 treatment for 72 h. β-actin was used as a loading control. **c** Cell viability of wild type HONE1 and CNE2 cells with (red) or without (blue) T-96 treatment by CCK-8 assay. All data represent the mean ± SD (*n* = 4, **P < 0.01, ***P < 0.001, ****P < 0.0001). **d** SA-β gal staining (left panel) of wild type HONE1 and CNE2 cells with or without doxycycline treatment for 7 days. Red arrows represent SA-β gal-positive cells. Scale bar = 50 μm. Dot plots (right panel) show quantification of the frequency of SA-β gal-positive cells in each vision. All data represent the mean ± SD (*n* = 4, ***P < 0.001, ****P < 0.0001). **e** Summary of the present study. Retinoic acid (RA) is balanced to low level concentration in SOX1 low NPC cells, while SOX1 induces RA accumulation within NPC cells, promoting cell differentiation (upper panel). For treatment strategy, targeting at UGT2B7 induces NPC cell differentiation, which is associated with RA metabolism.

UGTs glucuronidate RA and disrupt its biological activity. SOX1 might prevent the glucuronidation of RA by reducing the expression of UGTs and eventually lead to the accumulation of RA in cells to promote differentiation. Then, HONE1 and CNE2 cells were treated with T-96, an inhibitor of UGT1A6 and UGT2B7, and the cellular morphology was transformed into differentiated status (Fig. 6a). Western blot also revealed that the relevant differentiation markers, KRT5 and KRT13, were all decreased (Fig. 6b). T-96 could also inhibit cell proliferation and induce senescence (Fig. 6c, d). Thus, inhibition of UGTs led to differentiation of NPC cells.

## Discussion

Our new data provided evidences to suggest a novel therapeutic strategy for application of differentiation therapy in NPC by revealing several new findings: (1) SOX1 was involved in promoting differentiation of NPC cells depending on its transcriptional function. (2) Low expression of SOX1 was associated with high expression of keratins, similar to clinical NPC tissues. (3) SOX1 promoted differentiation of NPC cells through increasing retinoids accumulation within or outside NPC cells, which was associated with decreasing expression of UGTs. (4) T-96, a small molecular inhibitor of UGT1A6 and UGT2B7, induced cell differentiation and could be used in differentiation therapy for NPC.

In the present treatment of NPC, radiotherapy displays favorable therapeutic outcomes for early stage patients. For locoregionally advanced NPC patients, platinum-based concurrent chemoradiotherapy is the standard therapeutic regimen. Induction chemotherapy together with chemoradiotherapy could significantly improve progression-free survival and overall survival compared with chemoradiotherapy alone^2^. However, local failure and distant metastasis are still the major challenges for the poor survival of advanced NPC patients, and other therapeutics are needed to be developed for better outcome.

Differentiation therapy has achieved promising results in certain types of cancers, especially in leukemia^3,4^. More than 95% NPC are type III undifferentiated carcinoma. Patients diagnosed with undifferentiated carcinoma have a poor prognosis and high mortality rate, which calls for the demand of differentiation therapy. Our previous study found that both NPC cell lines and NPC tissues showed decreased expression of SOX1 at the mRNA and protein levels, which was associated with promoter hypermethylation. Ectopic expression of SOX1 repressed NPC cell proliferation, migration, and induced cell differentiation^16^. However, the mechanism SOX1-induced cell differentiation remains unclear. Here, we found that the deletion or mutation of either DNA-binding domain or transcription activation domain of SOX1 impaired the differentiation of NPC cells (Fig. 2 and Supplementary Fig. 2). Thus, SOX1 promoting differentiation depended on its transcriptional function.

Besides, we found that a global keratins high expression event in NPC tissues compared to normal tissues (Fig. 3a). Ectopic expression of SOX1 in NPC cells downregulated the expression of keratins, including KRT5, KRT13, and KRT19, which was similar to the gene expression profiles between NPC tissues and normal tissues (Fig. 3a–d). As keratins family proteins are potential markers for defining differentiation status of NPC cells^26,27^, these results furtherly demonstrated SOX1-induced NPC cell differentiation.

Moreover, we cultured cells with CM obtained from cells with SOX1 overexpression. Cells treated with CM showed decreased keratins, suggesting that components in CM promoted differentiation of NPC cells (Fig. 4c). LC/MS is currently the most widely used method of determining metabolic phenotypes via both untargeted and targeted analysis. Therefore, in order to determine components that induced the differentiation of NPC cells, untargeted metabolic LC/MS for CM and cells were performed, and high level of retinoids was detected both in CM and cells with SOX1 overexpression (Fig. 4d, e). We and others reported that ATRA inhibited proliferation, and induced differentiation of NPC cells^18,28^. Our results showed that cells treated with RAce or RA presented reduced keratins expression, declined proliferation and colony formation (Fig. 4f–h). Hence, retinoid metabolic pathway was involved in SOX1-induced cell differentiation. Previously, it was reported that SOX2 could affect glycolysis by regulating GLUT1, therefore contributing to the disease progression of squamous cell carcinoma^29^. But it remains elusive whether SOX1 could regulate metabolic-related genes and pathways.

Genes in regulating retinoid metabolic pathway were screened. The expression level of UGTs was significantly reduced in poorly differentiated cells after doxycycline induction (Fig. 5d). CYP26s or UGTs were reported to clear RA by hydroxylation or glucuronidation^21,22,30^. UGTs catalyze the transfer of glucuronic acid to lipophilic substrates, and convert them into hydrophilic compounds that can be excreted from cells. Crystal structure of UGT2B7 is recently identified, which catalyzes the covalent addition of glucuronic acid sugar moieties to a host of therapeutics and environmental toxins^31^. UGT2B7 has also been showed as responsible for the glucuronidation of ATRA with high catalytic efficiency^30^. The drug-induced UGTs expression has been repeatedly observed after exposure of cancer cells to anti-cancer drugs. UGT2B7 expression was demonstrated in human melanocytes, but not cell lines derived from metastatic melanomas. Treatment of these cell lines with anti-cancer agents, including vemurafenib, induced expression of UGT2B7^32^. Epirubicin upregulated UGT2B7 expression in hepatocellular carcinoma HepG2 and Huh7 cells, promoting its own detoxification via the p53-mediated pathway^33^. Besides, a further study showed a similar induction of UGT2B7 by several other cytotoxic drugs, including three anthracyclines (doxorubicin, daunorubicin, and idarubicin) and six nonanthracyclines (mitomycin C, 5-fluorouracil, camptothecin, 7-ethyl-10-hydroxycamptothecin, topotecan, and etoposide) in hepatocellular carcinoma cells^34^. Thus, drug-induced UGT2B7 activity in cancer cells affected the therapeutic efficacy of cytotoxic drugs and noncytotoxic drugs, which were UGT2B7 substrates. It was shown that ATRA-induced transcription via a RARE in the presence of RARs^35^. Although glucuronidation of ATRA might produce retinoyl β-glucuronide or other metabolites, retinoyl β-glucuronide binds to neither RARs nor CRABPs^36,37^. Thus, RA glucuronidation might result in attenuating the differentiation induced by SOX1 in NPC cells.

T-96 was reported to inhibit UGT1A6 (Ki = 0.6 μM) and UGT2B7 (Ki = 17.3 μM)^38^. It was originally used as an immunosuppressive agent, which significantly inhibited the activation of NF-κB^39^. Later studies reported that it also had anti-cancer effects. T-96 inhibited cell proliferation and induced cell apoptosis through downregulating the expression of MCL1 in melanoma cells^40^. T-96 inhibited triple-negative breast cancer invasion by blocking the canonical and non-canonical TGF-β signaling pathways^41^. And it suppressed glioma growth by regulating the miR-30e-5p/MYBL2 axis^42^. These results demonstrated that T-96 might act as a promising agent for the treatment of cancers. Here, we found T-96 promoted the differentiation of NPC cells (Fig. 6a, b). Moreover, T-96 significantly inhibited HONE1 and CNE2 cell proliferation and induced cell senescence (Fig. 6c, d). Therefore, UGTs could be used as targets for differentiation therapy in NPC treatment.

Collectively, we showed that SOX1 promoted NPC differentiation by enhancing retinoids accumulation through deregulating UGTs expression. Our data provide compelling biomolecular basis to study UGTs as targets for differentiation therapy and reveal the potential use of its inhibitor T-96 for NPC treatment (Fig. 6e).

## Supplementary information

supplemental Figure 1

supplemental Figure 2

supplemental Figure 3

supplemental Figure 4

supplemental Figure 5

supplemental Figure 6

supplemental Figure 7

supplemental Figure 8

Supplemental figure legends

Supplementary Table S1

Supplementary Table S2

Supplementary Table S3

Supplementary Table S4
