# Supplementary figures and images for: SOX1 promotes differentiation of nasopharyngeal carcinoma cells by activating retinoid metabolic pathway
Source: Cell Death Dis. 2020 May 7;11(5):331. doi: 10.1038/s41419-020-2513-1 (PMC7206110; doi:10.1038/s41419-020-2513-1)

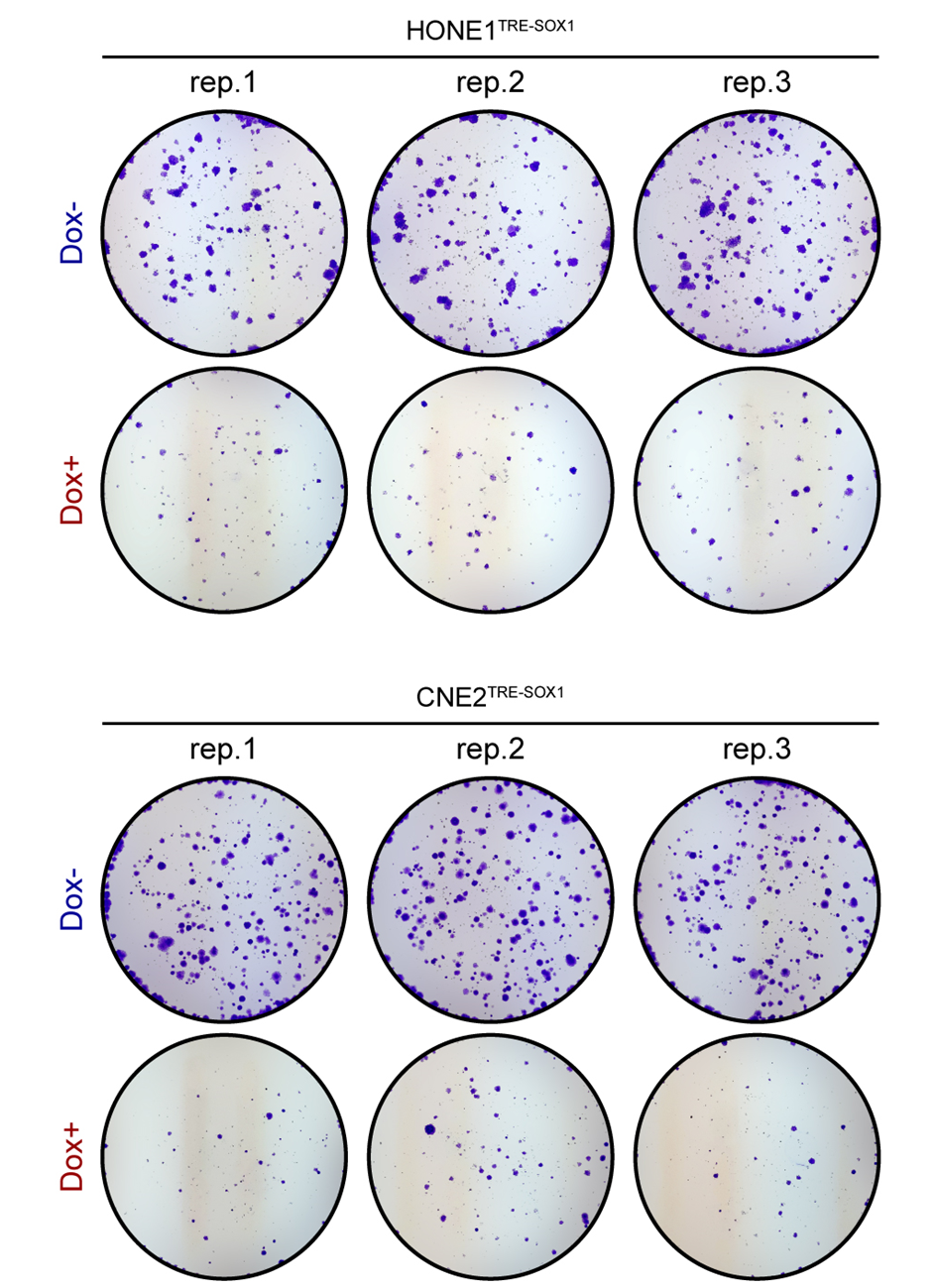

Supplement: Supplementary file 1 — supplemental Figure 1 [file 41419_2020_2513_MOESM1_ESM.tif]

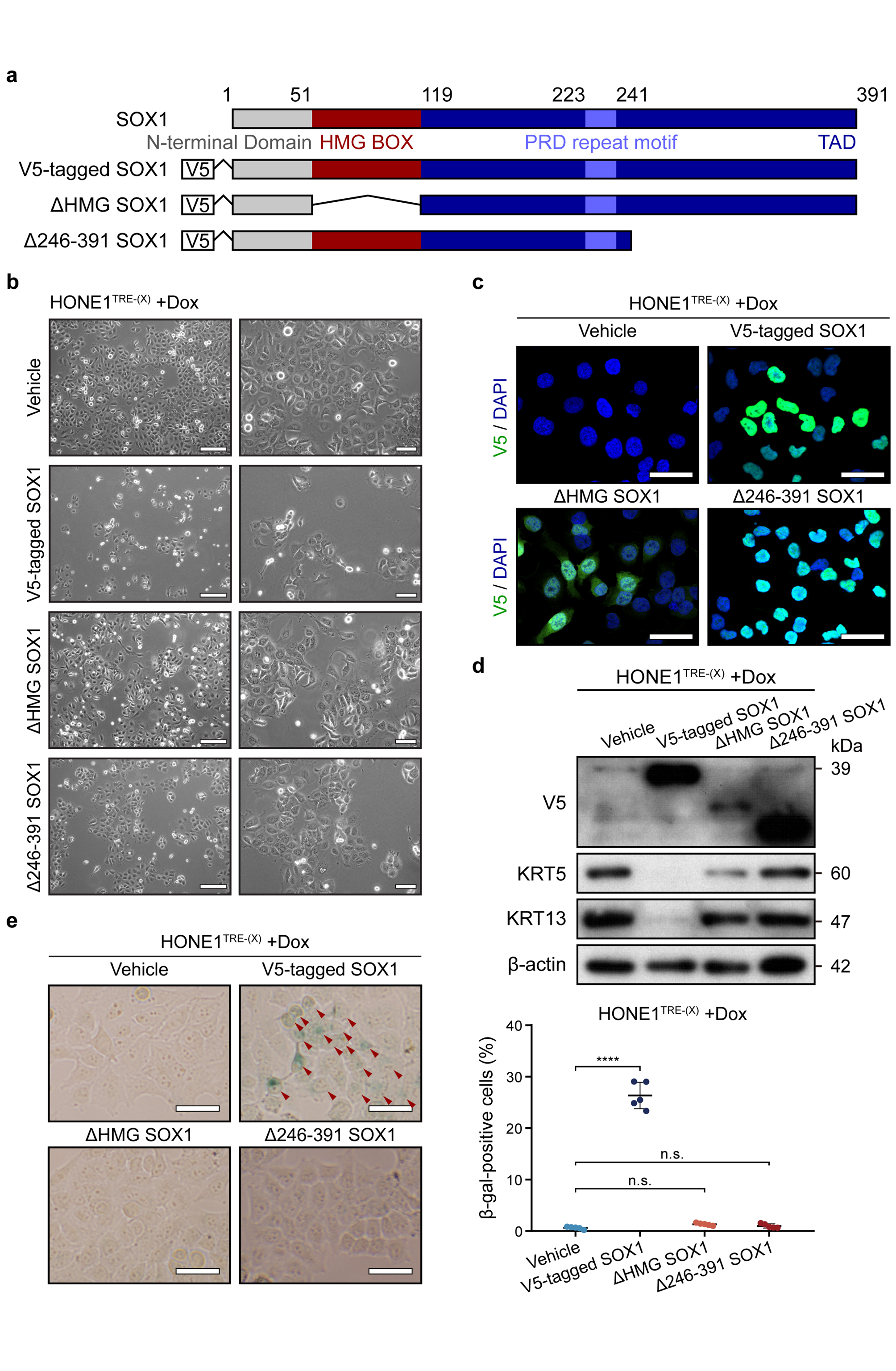

Supplement: Supplementary file 2 — supplemental Figure 2 [file 41419_2020_2513_MOESM2_ESM.tif]

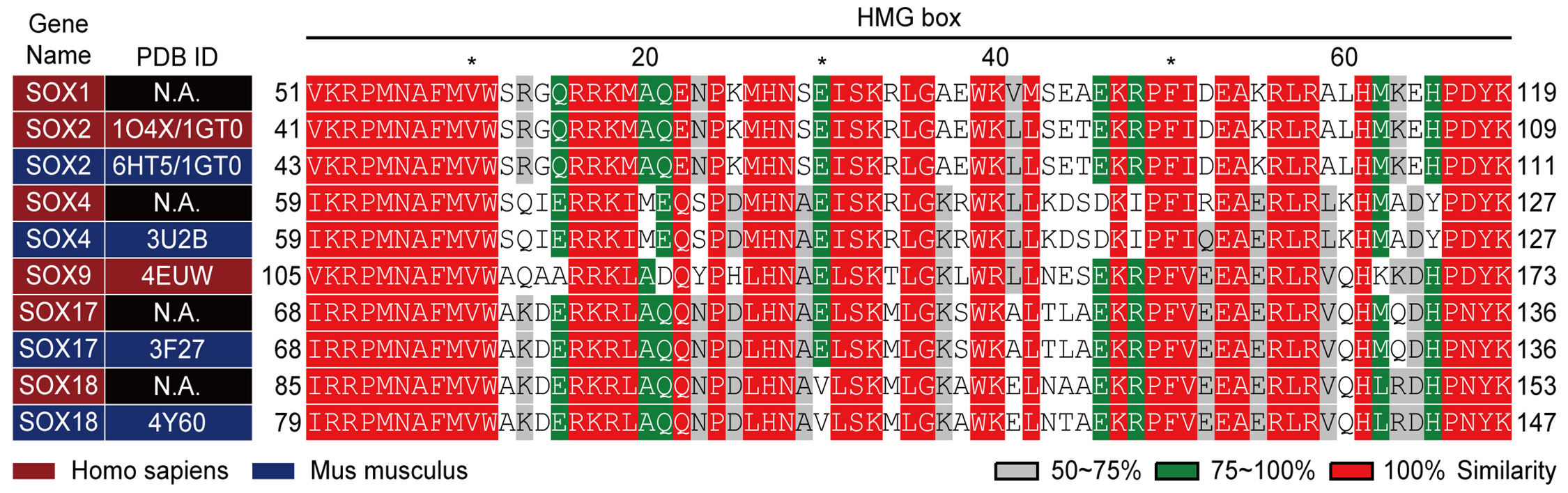

Supplement: Supplementary file 3 — supplemental Figure 3 [file 41419_2020_2513_MOESM3_ESM.tif]

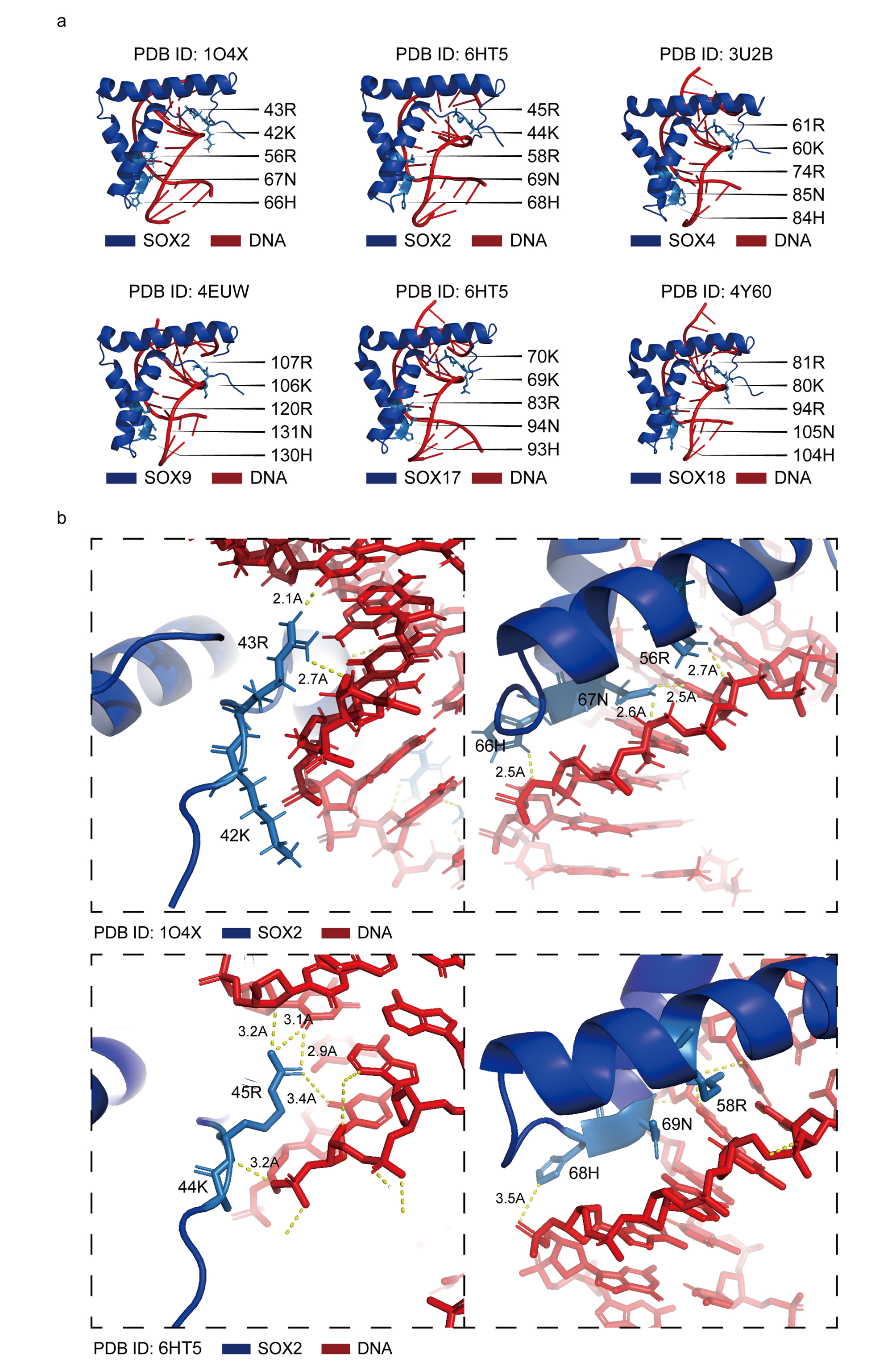

Supplement: Supplementary file 4 — supplemental Figure 4 [file 41419_2020_2513_MOESM4_ESM.tif]

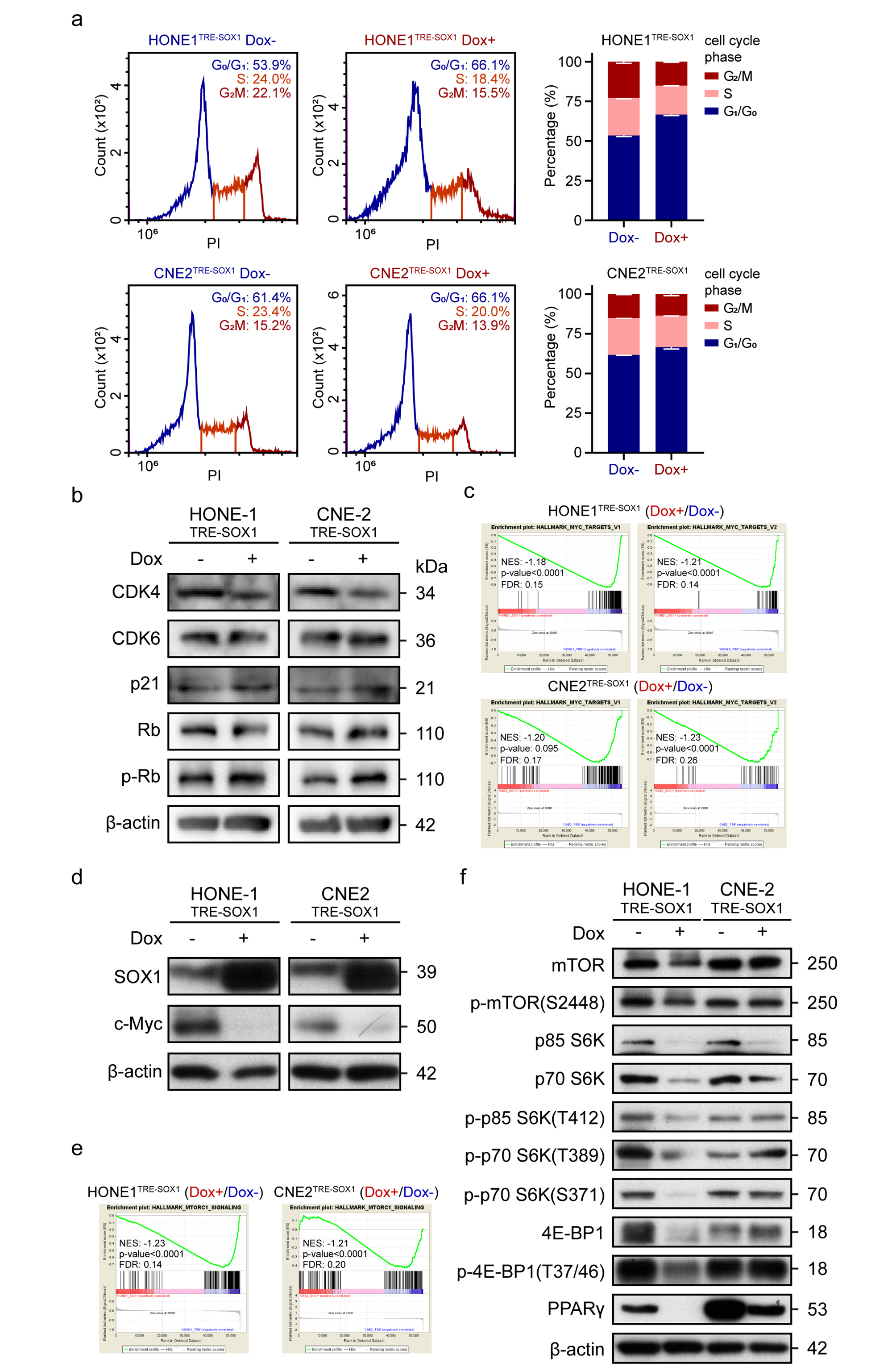

Supplement: Supplementary file 5 — supplemental Figure 5 [file 41419_2020_2513_MOESM5_ESM.tif]

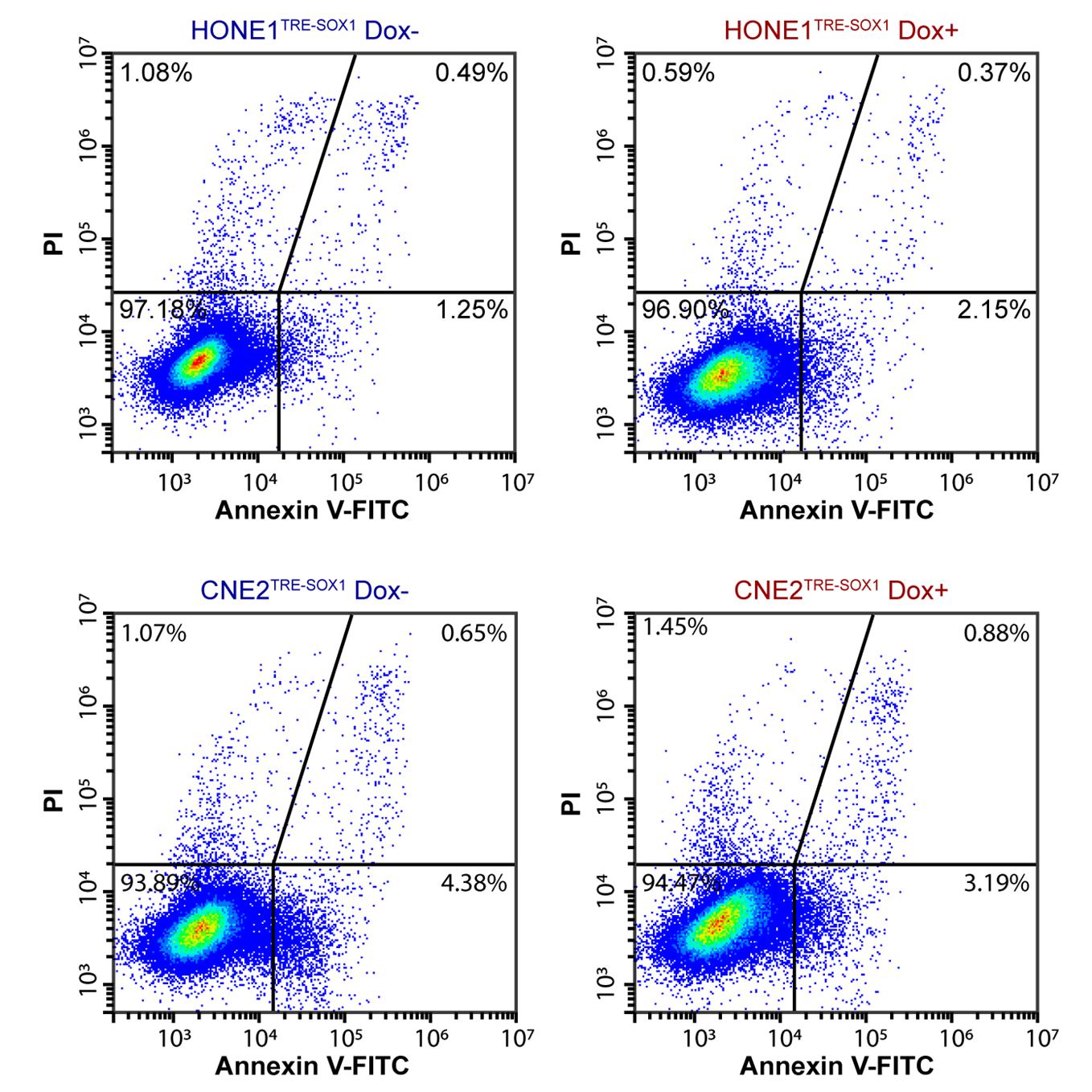

Supplement: Supplementary file 6 — supplemental Figure 6 [file 41419_2020_2513_MOESM6_ESM.tif]

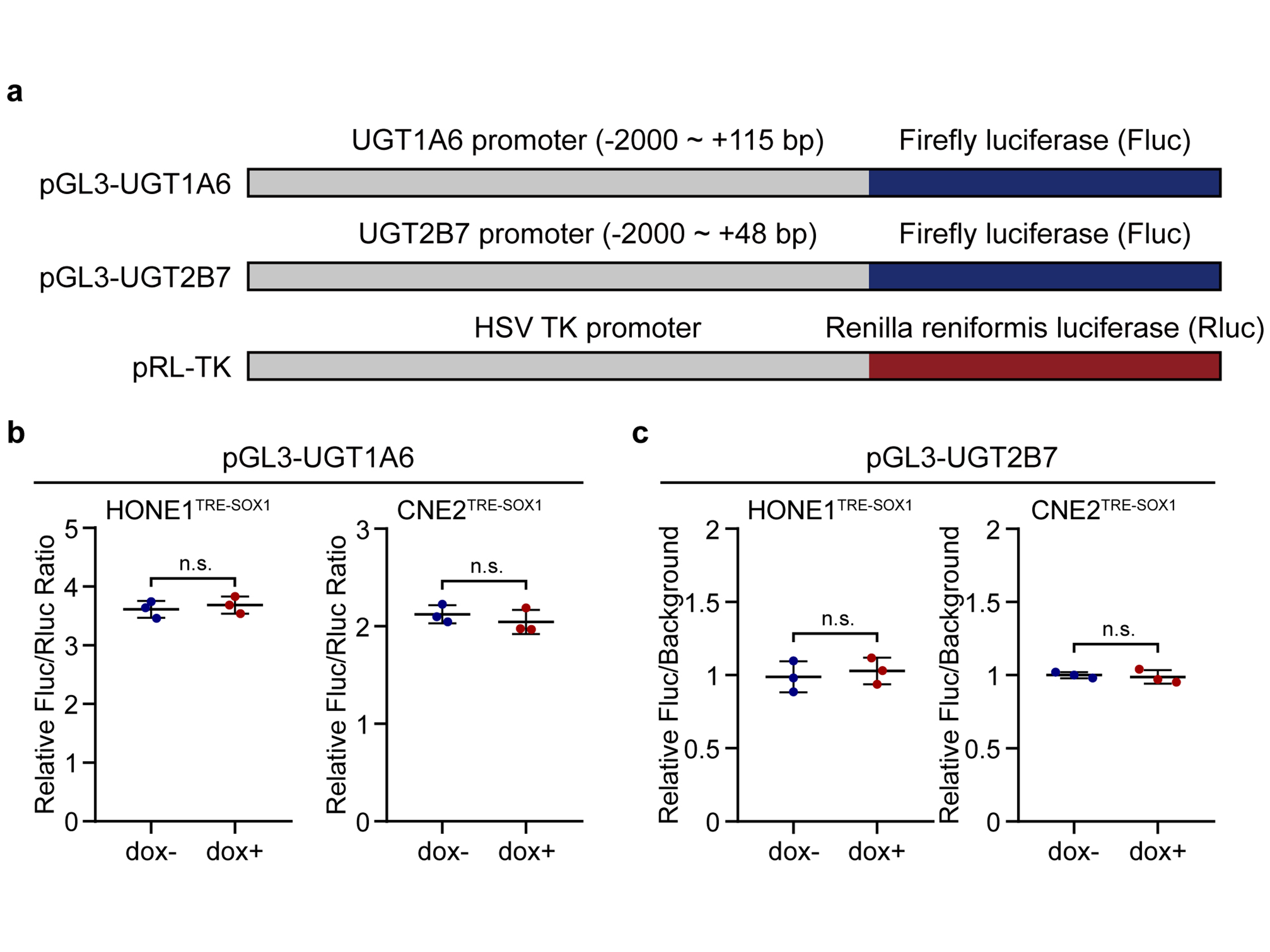

Supplement: Supplementary file 7 — supplemental Figure 7 [file 41419_2020_2513_MOESM7_ESM.tif]

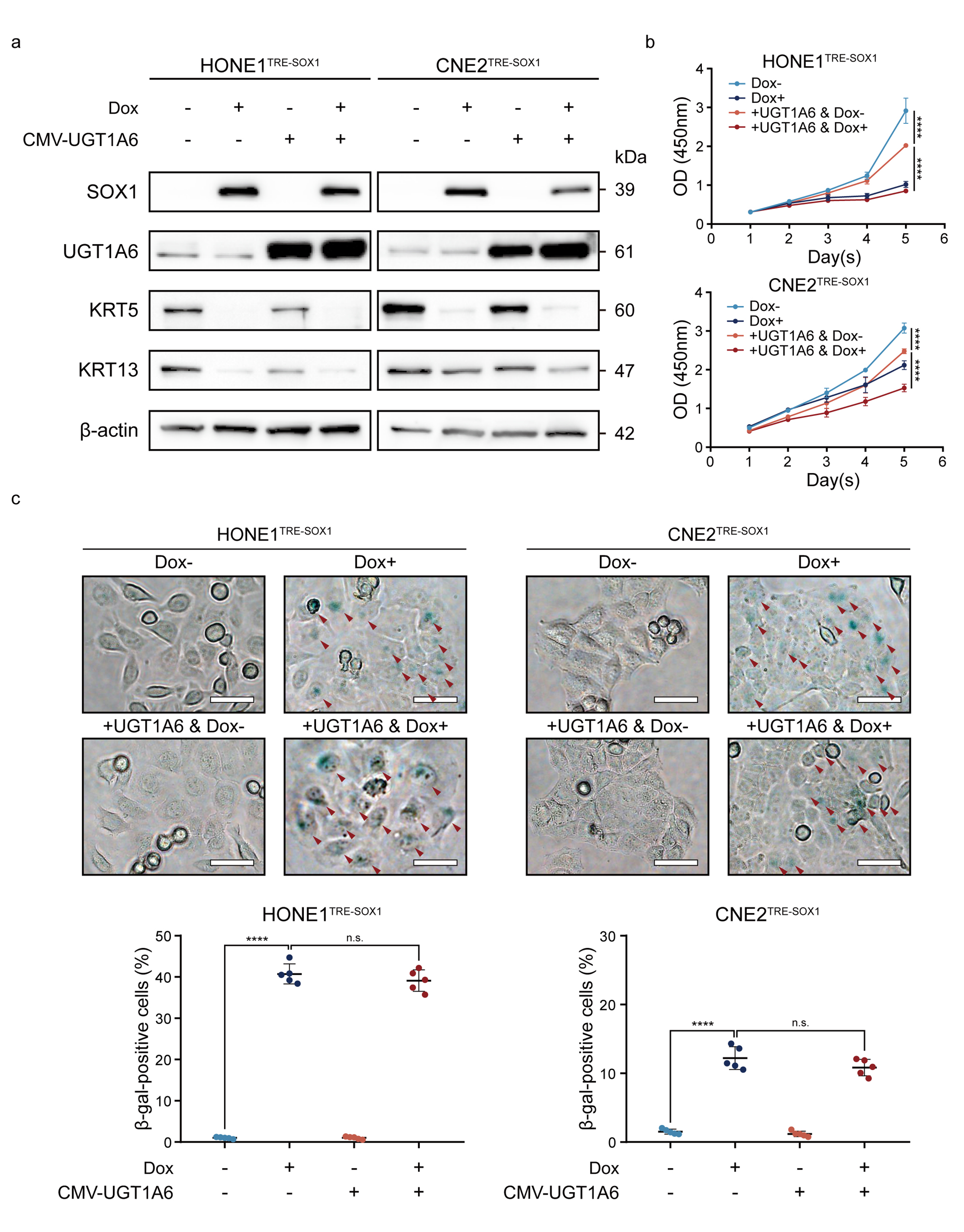

Supplement: Supplementary file 8 — supplemental Figure 8 [file 41419_2020_2513_MOESM8_ESM.tif]
