# Supplementary material for: SOX1 promotes differentiation of nasopharyngeal carcinoma cells by activating retinoid metabolic pathway
Source: Cell Death Dis. 2020 May 7;11(5):331. doi: 10.1038/s41419-020-2513-1 (PMC7206110; doi:10.1038/s41419-020-2513-1)
Supplement: Supplementary file 9 — Supplemental figure legends [file 41419_2020_2513_MOESM9_ESM.docx]

**Supplementary Figure legends**

**Supplementary Figure 1. Photography of colony formation assay.**

Colony formation assay of HONE1^TRE-SOX1^ and CNE2^TRE-SOX1^ cells with or without doxycycline treatment for 8 days. Each group displays three independent experiments.

**Supplementary Figure 2. SOX1 promotes NPC cell differentiation depending on its transcriptional function.**

(a) Schematic of full length and truncated SOX1 proteins used in this study. All proteins are fused with V5 tag at N-terminus. (b) Morphology of HONE1^TRE-(X)^ cells (X stand for vehicle, V5-SOX1, V5-SOX1^ΔHMG^ or V5-SOX1^Δ246-391^) under doxycycline treatment for 3 days. Scale bar = 100µm (left) / 50µm (right). (c) Confocal immunofluorescence for V5 (green) and DAPI (blue) in HONE1^TRE-(X)^ cells under doxycycline treatment for 3 days. Scale bar = 50 μm. (d) Western blot analysis of V5-tagged proteins, KRT5, KRT13 and β-actin expression in HONE1^TRE-(X)^ cells under doxycycline treatment for 3 days. β-actin was used as a control. (e) SA-β gal staining (left panel) of HONE1^TRE-(X)^ cells under doxycycline treatment for 7 days. Red arrows represent SA-β gal-positive cells. Scale bar = 50μm. Dot plots (right panel) show quantification of the frequency of SA-β gal-positive cells in each vision. All data represent the mean ± SD (n=5, n.s.: P>0.05, ****P < 0.0001).

**Supplementary Figure 3. Sequence alignment of the HMG box domain for different SOX superfamily members.**

Amino acid sequences of SOX1, SOX2, SOX4, SOX9, SOX17 and SOX18 HMG-BOX domain. Conventional motifs/residues are highlighted in red and green. (PDB ID: Protein Data Bank code).

**Supplementary Figure 4. Structure comparison of DNA binding domain of SOX subfamily members.**

(a) Structural comparison of the DNA binding domains (HMG-BOX) of SOX2 (PDB ID: 1O4X, homo sapiens, 6HT5, musculus), SOX4 (3U2B, musculus), SOX9 (4EUW, homo sapiens), SOX17 (3F27, musculus) and SOX18 (4Y60, musculus). Five amino acids which are near grooves of DNA in each structure are labelled. (b) The DNA binding sites in HMG box of SOX2. Upper, homo sapiens; lower, musculus.

**Supplementary Figure 5. SOX1 alters cell cycle in NPC cells.**

(a) Flow cytometry of HONE1^TRE-SOX1^ and CNE2^TRE-SOX1^ cells with or without doxycycline treatment for 3 days. PI dye was used to divide cells to three cell cycle phase: G0/G1, S and G2/M phase (left panel). Quantitative proportion of cell cycle phase was displayed (right panel). All data represent the mean ± SD. (b) Western blot analysis of cell cycle associated proteins in HONE1^TRE-SOX1^ and CNE2^TRE-SOX1^ cells with or without doxycycline treatment for 96 h. β-actin was used as a loading control. (c) GSEA of SOX1 (Dox+) vs control (Dox-) in HONE1^TRE-SOX1^ and CNE2^TRE-SOX1^ cells using ‘Hallmark MYC targets v1’ and ‘Hallmark MYC targets v2’ gene sets. (NES: normalized enrichment score, FDR: false discovery rate) (d) Western blot analysis of c-Myc and SOX1 in HONE1^TRE-SOX1^ and CNE2^TRE-SOX1^ cells with or without doxycycline treatment for 96 h. β-actin was used as a loading control. (e) GSEA of SOX1 (Dox+) vs control (Dox-) in HONE1^TRE-SOX1^ and CNE2^TRE-SOX1^ cells using ‘Hallmark mTORC1 signaling’ gene sets. (NES: normalized enrichment score, FDR: false discovery rate) (f) Western blot analysis of mTOR1 pathway associated proteins in HONE1^TRE-SOX1^ and CNE2^TRE-SOX1^ cells with or without doxycycline treatment for 96 h. β-actin was used as a loading control.

**Supplementary Figure 6. SOX1 does not induce obvious cell apoptosis in NPC cells.**

Flow cytometry of HONE1^TRE-SOX1^ and CNE2^TRE-SOX1^ cells with or without doxycycline treatment for 3 days. FITC labelled Annexin V and PI double dyes were used to display early apoptosis (Annexin V-high/PI-low) and late apoptosis (Annexin V-high/PI-high).

**Supplementary Figure 7. Luciferase assay of UGT1A6- and UGT2B7-2000bp up-stream elements.**

(a) Schematics of promoters fused firefly luciferase (Fluc) or renilla reniformis luciferase (Rluc). 2000bp up-stream DNA sequence of UGT1A6 and UGT2B7 were inserted and fused in front of Fluc. Rluc was used as control. Dual-luciferase assay of HONE1^TRE-SOX1^ and CNE2^TRE-SOX1^ cells with or without doxycycline treatment for 3 days was shown. Relative Fluc/Rluc Ratio represented transcriptional activity of UGT1A6-2000bp (b) or UGT2B7-2000bp (c) up-stream element regulated by SOX1.

**Supplementary Figure 8. UGT1A6 does not rescue NPC cell differentiation promoted by SOX1.**

(a) Western blot analysis of SOX1, UGT1A6, KRT5, KRT13 and β-actin expression in HONE1^TRE-SOX1,^ CNE2^TRE-SOX1^ as well as UGT1A6 overexpressed HONE1^TRE-SOX1^ and CNE2^TRE-SOX1^ cells under doxycycline treatment for 4 days. β-actin was used as a control. (b) Cell viability of HONE1^TRE-SOX1^, CNE2^TRE-SOX1^ as well as UGT1A6 overexpressed HONE1^TRE-SOX1^ and CNE2^TRE-SOX1^ cells with or without doxycycline treatment by CCK-8 assay. All data represent the mean ± SD (n=4, ****P < 0.0001). (c) SA-β gal staining (upper panel) of HONE1^TRE-SOX1^, CNE2^TRE-SOX1^ as well as UGT1A6 overexpressed HONE1^TRE-SOX1^ and CNE2^TRE-SOX1^ cells under doxycycline treatment for 7 days. Red arrows represent SA-β gal-positive cells. Scale bar = 50μm. Dot plots (lower panel) show quantification of the frequency of SA-β gal-positive cells in each vision. All data represent the mean ± SD (n=5, n.s.: P>0.05, ****P < 0.0001).
