# Supplementary material for: SOX1 promotes differentiation of nasopharyngeal carcinoma cells by activating retinoid metabolic pathway
Source: Cell Death Dis. 2020 May 7;11(5):331. doi: 10.1038/s41419-020-2513-1 (PMC7206110; doi:10.1038/s41419-020-2513-1)
Supplement: Supplementary file 10 — Supplementary Table S1 [file 41419_2020_2513_MOESM10_ESM.docx]

| **Supplementary Table S1. Primer sequences for gene cloning and plasmid construction** | | |
| --- | --- | --- |
| **Insertion name (X)** | **Forward primer (5'-3')** | **Reverse primer (5'-3')** |
| SOX1 | CTTTTGTCTTATACTTGGATCGCCACCATGTACAGCATGATG | CTCCCCTACCCGGTAGAATTCTAGATGTGCGTCAGGGGCACCG |
| SOX1 mut_2 fra.1 | CTTTTGTCTTATACTTGGATCGCCACCATGTACAGCATGATG | ACCATGAAGGCGTTCATGGGGTCTTTGACCCGGTCCTGGTTG |
| SOX1 mut_2 fra.2 | CAACCAGGACCGGGTCAAAGACCCCATGAACGCCTTCATGGT | AGGCGCTTGCTGATCTCCGAGGCGTGCATCTTGGGGTTCTCCT |
| SOX1 mut_2 fra.3 | AGGAGAACCCCAAGATGCACGCCTCGGAGATCAGCAAGCGCCT | CTCCCCTACCCGGTAGAATTCTAGATGTGCGTCAGGGGCACCG |
| V5-SOX1 | TTTTGTCTTATACTTGGATCATGGGTAAGCCTATCCCTAACCCTCTCCTCGGTCTCGATTCTACGATGTACAGCATGATGATGGA | CTCCCCTACCCGGTAGAATTCTAGATGTGCGTCAGGGGCACCGT |
| ΔHMG-SOX1 fra.1 | TTTTGTCTTATACTTGGATCATGGGTAAGCCTATCCCTAACCCTCTCCTCGGTCTCGATTCTACGATGTACAGCATGATGATGGA | GCGGCGCGGCCGGTACCGGTCCTGGTTGGCCTTG |
| ΔHMG-SOX1 fra.2 | TACCGGCCGCGCCGCAAGACCAA | CTCCCCTACCCGGTAGAATTCTAGATGTGCGTCAGGGGCACCGT |
| Δ246-391 SOX1 | TTTTGTCTTATACTTGGATCATGGGTAAGCCTATCCCTAACCCTCTCCTCGGTCTCGATTCTACGATGTACAGCATGATGATGGA | CTCCCCTACCCGGTAGAATTCTAGGGCTGCGGGTTGTGC |
| UGT1A6-promoter | GCGTGCTAGCCCGGGCTCGAATGGTATGCATAGTGATGTT | CAGTACCGGAATGCCAAGCTCCTGGAAAGAGTTAAAGTAAC |
| UGT2B7-promoter | GCGTGCTAGCCCGGGCTCGAGTTTACACAATGCTATAGCA | CAGTACCGGAATGCCAAGCTCCTGGTGCAATGCAATGCTT |
| UGT1A6 | GGATCTATTTCCGGTGAATTATGGCCTGCCTCCTTCGCTCAT | GGAGGGAGAGGGGCGGGATCTCAATGGGTCTTGGATTTGTG |
| UGT2B7 | GGATCTATTTCCGGTGAATTATGTCTGTGAAATGGACTTC | GGAGGGAGAGGGGCGGGATCCTAATCATTTTTTCCCTTCT |
