# Supplementary material for: SOX1 promotes differentiation of nasopharyngeal carcinoma cells by activating retinoid metabolic pathway
Source: Cell Death Dis. 2020 May 7;11(5):331. doi: 10.1038/s41419-020-2513-1 (PMC7206110; doi:10.1038/s41419-020-2513-1)
Supplement: Supplementary file 11 — Supplementary Table S2 [file 41419_2020_2513_MOESM11_ESM.docx]

| **Supplementary Table S2. Primers for qPCR analysis of 5 keratin genes and internal control gene.** | | | | | | | |
| --- | --- | --- | --- | --- | --- | --- | --- |
| **Gene name** | | **GenBank Accession No.** | **Forward primer (5'-3')** | | | **Reverse primer (5'-3')** | |
| **5 keratin genes** | | | | | | | |
| KRT5 | | NM_000424 | GGCGAGGAATGCAGACTCAG | | | GTAGCTTCCACTGCTACCTCC | |
| KRT13 | | NM_153490 | CCCCAGGCATTGACCTGAC | | | GTGTTGGTAGACACCTCCTTG | |
| KRT14 | | NM_000526 | CCAGCTCAGCATGAAAGCATC | | | CGTGCACATCCATGACCTTG | |
| KRT19 | | NM_002276 | TGGAGATGCAGATCGAAGGC | | | CTTCAGTCCGGCTGGTGAAC | |
| KRT80 | | NM_001081492 | TCAGCTGAAGAAGGACCTGGAT | | | GGTCACCGACACATCCTTCA | |
| **Internal control gene** | | | | | | |  |
| ACTB | NM_001101 | | | TTGCCGACAGGATGCAGAAGGA | AGGTGGACAGCGAGGCCAGGAT | |  |
