# Supplementary material for: SOX1 promotes differentiation of nasopharyngeal carcinoma cells by activating retinoid metabolic pathway
Source: Cell Death Dis. 2020 May 7;11(5):331. doi: 10.1038/s41419-020-2513-1 (PMC7206110; doi:10.1038/s41419-020-2513-1)
Supplement: Supplementary file 12 — Supplementary Table S3 [file 41419_2020_2513_MOESM12_ESM.docx]

| **Supplementary Table S3. Primers for qPCR analysis of 9 retinoic acid signaling genes and internal control gene.** | | | |
| --- | --- | --- | --- |
| **Gene name** | **GenBank Accession No.** | **Forward primer (5'-3')** | **Reverse primer (5'-3')** |
| **9 retinoic acid signaling genes** | | | |
| STRA6 | NM_001199040 | TCGCTGTCAATCCTTGTGCT | TGTCCCCAGCCAAGAAATCC |
| CRABP1 | NM_004378 | ACGCAAGTGCAGGAGTTTAG | CGGGTCCAGTAGGTTTTGGG |
| CRABP2 | NM_001878 | GCCCTGTAAGAGCCTGGTGA | AGTTCTCTGGTCCACGAGGT |
| RARA | NM_001024809 | GCCTGGACATCCTGATCCTG | TCCGCACGTAGACCTTTAGC |
| RARB | NM_000965 | CGTGGAGTTTGCTAAACGTCT | TGGTGTCTTGTTCTGGGGTAT |
| RARG | NM_001243732 | ATGCTGCGTATCTGCACAAG | AGGCAAAGACAAGGTCTGTGA |
| RXRA | NM_002957 | GACGGAGCTTGTGTCCAAGAT | AGTCAGGGTTAAAGAGGACGAT |
| RXRB | NM_021976 | GCAGCCCAAATGACCCTGT | CCCGCAGCAATATGACCTGA |
| RXRG | NM_001256571 | CGGGCAGGGTGGAATGAAT | CTTGGTGTAGGCCTCAAGGG |
| **Internal control gene** | | | |
| ACTB | NM_001101 | TTGCCGACAGGATGCAGAAGGA | AGGTGGACAGCGAGGCCAGGAT |
