# Supplementary material for: SOX1 promotes differentiation of nasopharyngeal carcinoma cells by activating retinoid metabolic pathway
Source: Cell Death Dis. 2020 May 7;11(5):331. doi: 10.1038/s41419-020-2513-1 (PMC7206110; doi:10.1038/s41419-020-2513-1)
Supplement: Supplementary file 13 — Supplementary Table S4 [file 41419_2020_2513_MOESM13_ESM.docx]

| **Supplementary Table S4. Primers for qPCR analysis of 10 retinoic acid metabolism genes and internal control gene.** | | | |
| --- | --- | --- | --- |
| **Gene name** | **GenBank Accession No.** | **Forward primer (5'-3')** | **Reverse primer (5'-3')** |
| **10 retinoic acid metabolism genes** | | | |
| LRAT | NM_004744 | CGCCTACGGAGCTAACATCC | CTGATCGGGGTGCCATATCTG |
| CYP26A1 | NM_000783 | GAATGCTACGTGCCGGTGAT | CATGGCGATTCGGAACATGAG |
| CYP26B1 | NM_019885 | GGCAACGTGTTCAAGACGC | TGCTCGCCCATGAGGATCT |
| CYP26C1 | NM_183374 | CACACTGCTAGGTGCGGTC | TGGAGGCGTCGTAGACTGAG |
| UGT1A (total) | not available | TGGAACCCGACCATCGAATC | CATCGGGTGACCAAGCAGAT |
| UGT1A1 | NM_000463 | CTGTCTCTGCCCACTGTATTCT | TCTGTGAAAAGGCAATGAGCAT |
| UGT1A6 | NM_205862 | GAGGAAAGACTTGTCTCAGGAAT | GGTGATAAAGGCACGGGTCA |
| UGT1A9 | NM_021027 | TGATCTCTACAGCCACACATC | TTCAAATTCCATAGGCAACGGC |
| UGT2B7 | NM_001349568 | GGGAAAGCTGACGTATGGCT | CCATTTCCTTAGGCAGGGGT |
| UGT8 | NM_003360 | CAGCCTGTGGATGCTGTGTA | TTGGGTCCAGAAAACCTCCA |
| **Internal control gene** | | | |
| ACTB | NM_001101 | TTGCCGACAGGATGCAGAAGGA | AGGTGGACAGCGAGGCCAGGAT |
